# COPD deaths attributable to ozone in 2019 and future projections using the WHO AQG 2021 in urban China

**DOI:** 10.1016/j.eehl.2022.11.002

**Published:** 2022-11-28

**Authors:** Mingyao Yao, Ying Hu, Ao Zhang, John S. Ji, Bin Zhao

**Affiliations:** a Department of Building Science, School of Architecture, Tsinghua University, Beijing 100084, China; b Vanke School of Public Health, Tsinghua University, Beijing 100084, China; c Beijing Key Laboratory of Indoor Air Quality Evaluation and Control, Tsinghua University, Beijing 100084, China

**Keywords:** Ozone, Environmental risk, Respiratory disease, Exposure, Mortality, Air Quality Guidelines

## Abstract

Chronic obstructive pulmonary disease (COPD) is an epidemic in China. Ozone is a possible risk factor of COPD, with ozone concentrations increasing in China, despite air pollution mitigation measures that reduced particulate matter. The WHO Air Quality Guidelines (AQG) recommendations in 2021 are a turning point that formally recognizes the crucial role of indoor air pollution. We aimed to investigate the premature COPD deaths attributable to ozone in 2019, taking the WHO AQG 2021 level into account to determine the gap to bridge ozone control in China. First, we assessed ozone exposures initiated from indoor and outdoor sources by gender and age groups in 344 cities under four scenarios: 2019 as a baseline, and outdoor ozone at WHO AQG 2021 level in 2019, 2030, and 2050, respectively. Subsequently, we estimated COPD deaths attributable to ozone. The results show that the COPD deaths attributable to ozone are 77,737 in 2019, and 527, 872, 1355 if the outdoor ozone concentration is reduced to the WHO AQG 2021 level in 2019 (counterfactual scenario), 2030, and 2050, respectively in urban China. The indoor ozone sources only contribute to less than 5% of COPD deaths. A gap of 68.5 μg/m^3^ for the highest seasonal ozone concentration should be bridged to meet the WHO AQG 2021 and avoid over 76 thousand (98%) COPD deaths in 2019 in urban China.

## Highlights

• COPD deaths attributable to indoor and outdoor ozone were estimated.
• Three control scenarios to meet WHO AQG 2021 in different years were considered.
• 98% of COPD deaths could be avoided by reducing ozone to WHO AQG 2021 level.
• Indoor ozone sources contribute less than 5% of COPD deaths.
• Population growth and aging increase the urgency of ozone control.

## Graphical abstract

**Figure:**
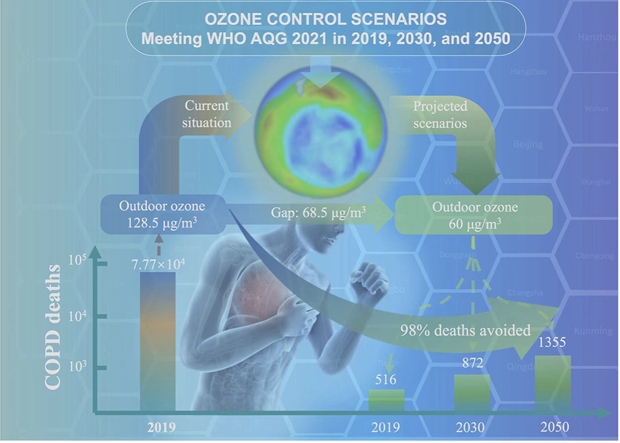

## 1 Introduction

Chronic obstructive pulmonary disease (COPD) is a common chronic lung disease and was associated with 3.23 million deaths in 2019 as the third-leading cause of death worldwide (https://www.who.int/data/global-health-estimates). China has a large share of COPD disease burden, with the prevalence of spirometry-defined COPD seeing an upward trajectory from 8.2% in 2002–2004 to 13.7% in 2012–2015 among people aged 40 years and older [1,2]. COPD causes persistent suffering in the population, marked by respiratory symptoms and airflow limitation caused by airway and alveolar abnormalities. The symptom onset is exacerbated with exposure to noxious particles or gases [3]. Studies have shown that ozone and particulate matter (PM_2.5_) are both acute and long-term risk factors to COPD [4,5], mainly including the acute exposure associated with disease onset and long-term exposures contributing to disease etiology. Ozone has been identified as a trigger of small airway functional impairment and dysfunction, as well as in cardiovascular disease [[6], [7], [8]]. Small airway abnormalities, in turn, limit the airflow in and out of the lungs or narrow the airways, further leading to the onset of COPD. As the only one ozone-attributable disease considered in the Global Burden of Disease Study (GBD) 2019 [9], COPD has attracted increasing attention. A large number of studies investigating the association between both short- and long-term ozone exposure and COPD in China have shown tremendous disease burden of COPD related to ozone [[10], [11], [12], [13], [14], [15], [16]].

The dramatic increase of ozone concentration in urban China has garnered public policy and health concerns. From 2014 to 2021, the annual-average outdoor ozone concentration increased by 56%, from 87.7 to 137 μg/m^3^ [17], while the concentration of the other air pollutants (inhalable particles, PM_2.5_, carbon monoxide, sulfur dioxide, and nitrogen dioxide) decreased. To further reduce global public health risks, the World Health Organization (WHO) has revised and published the Global Air Quality Guidelines (AQG) 2021. Based on existing evidence of the health effects between long-term ozone exposure and total and respiratory mortality, a seasonal average of peak ozone concentrations of 60 μg/m³ was added, with interim targets (IT) of 100 and 70 μg/m³ for IT-1 and IT-2, respectively [18]. While some parts of China have reduced PM_2.5_ exposures, the growing ozone pollution increases China’s existing challenges in controlling air pollution. A projection of ozone-attributable disease burden may help to develop ozone control strategies in the near future.

The morality burden of respiratory diseases attributable to ozone in 2050 and 2100 under different Representative Concentration Pathway scenarios [19], and Shared Socioeconomic Pathways (SSP) scenarios [20,21] have been estimated in China. However, these studies mainly considered the future speculative scenarios based on climate change and regarded ambient ozone as a proxy of human exposure to ozone. There is no estimation of source-specific disease burden of COPD attributable to ozone exposure nor the projection of COPD disease burden when outdoor ozone concentration meets WHO AQG 2021.

To elucidate the gap that needs to bridge to meet WHO AQG 2021, we estimated COPD deaths attributable to indoor and outdoor ozone sources in 2019 and further projected COPD deaths avoidable under different control scenarios where the outdoor ozone meets WHO AQG 2021 in urban China. In addition, the contribution of indoor ozone sources to the total COPD deaths was determined.

## 2 Materials and methods

### 2.1 Model framework and data sources

**Fig. 1:**
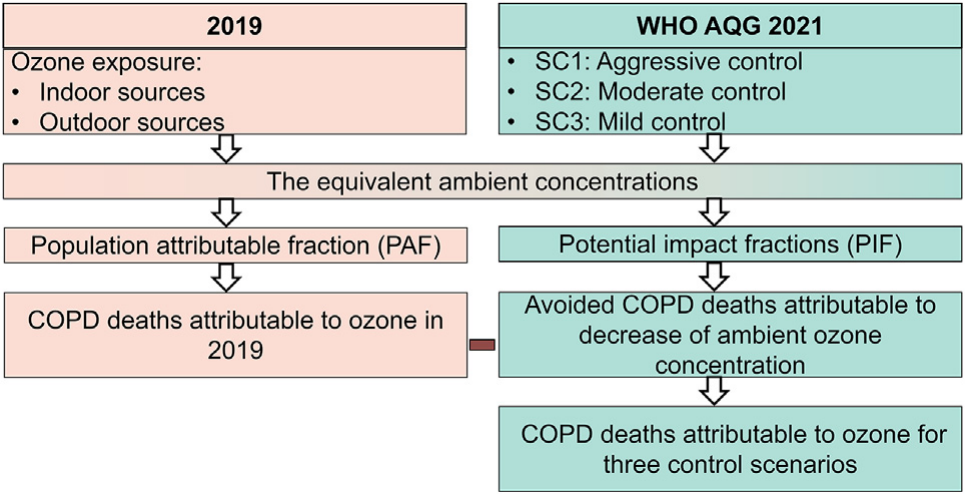
Scheme of the exposure concentration–death estimation model. COPD, chronic obstructive pulmonary disease; SC, scenario; WHO AQG, World Health Organization Global Air Quality Guidelines.

Exposure concentration, defined as the average concentration of ozone in the air people breathe during the studied period, is a more accurate indicator of the intake dose of ozone than ambient concentration. Therefore, we firstly estimated ozone exposure concentration initiated from indoor and outdoor ozone sources to reflect the actual exposure to ozone. Secondly, we calculated the equivalent ambient concentration of ozone by dividing the ozone exposure concentration through the ozone exposure factor (details see Sections 2.3, 2.4) [22], which was further used to estimate the relative risk (RR) following the GBD 2019 [9]. Based on the baseline death rates of COPD and the population of different ages and genders in different cities, we estimated the age-, gender-, city-, and source-specific COPD deaths in urban China in 2019 (baseline scenario) and when outdoor ozone reduced to WHO AQG 2021 in 2019 (aggressive control, a counterfactual scenario because 2019 has passed), 2030 (moderate control), and 2050 (mild control), respectively. A total of 19 age groups (0–1, 2–5, 6–10, 11–15, 16–20, 21–25, 26–30, 31–35, 36–40, 41–45, 46–50, 51–55, 56–60, 61–65, 66–70, 71–75, 76–80, 81–85, and >85) in 344 cities in 31 provinces were considered in this study. Fig. 1 shows the scheme of the exposure concentration–death estimation model.

In evaluating ozone exposure concentration from indoor and outdoor sources, the environmental parameters (outdoor ozone concentration, air change rates, penetration factor of ozone, room volume, ozone removal rates of indoor building/furniture and human surfaces, and emission rates of indoor ozone sources) and activity patterns (the time schedule of opening windows, operating ozone-emitting devices, and staying in indoors/outdoors) were needed. Hourly average outdoor ozone concentration in 2018 and 2019 was obtained from the National Urban Air Quality Real-time Publishing Platform (http://106.37.208.233:20035/), and data from 1497 fixed-site monitoring stations in 344 cities in China were gathered. The average hourly outdoor ozone concentration of all the monitors within one city was calculated to represent the ozone concentration of this city. The air infiltration rates and the natural ventilation rates (ventilation rates in residences with the windows open and no mechanical ventilation systems), penetration factor of ozone, and room volume have been published elsewhere [23]. Ozone removal rates of indoor building/furniture and human surfaces were calculated from the deposition velocities of ozone onto building/furniture and human surfaces indoors [24,25], details of which are shown in the section *Surface removal rates* in Supplementary materials (SM). The province-specific usages of ozone-emitting devices were obtained from a previous national survey which collected 1103 valid questionnaires from 31 provinces in China [26]. The retention rates of ozone-emitting devices are shown in Table S1. Emission rates of indoor ozone sources (Table S2) were obtained from a literature review [27]. Window opening schedules were determined from previous field measurements [28]. Schedules of ozone-emitting devices’ operating were determined from the national survey [26]. The determination of time being indoors/outdoors has been published elsewhere [23]. Details of the determination of activity patterns can be found in section *time schedules of different activities* in SM.

In evaluating the mortality burden of COPD attributable to ozone, the baseline death rates, population size, age structures, and concentration–response (C–R) relationship were needed. The baseline death rates of COPD in eastern, central, and western regions of China were obtained from the literature [29]. We calculated the ratio of age-standardized baseline death rates in 2019 to 2013 in the three regions and then obtained the province-specific baseline death rates in 2019 based on that in 2013 [[29], [30], [31]]. Tables S6 and S7 show the province-, age-, and gender-specific baseline death rate of COPD in urban China. Province-specific populations and proportion of different age groups were obtained from the National Bureau of Statistics of China [32] in 2019 and literature prediction, see details in section *age structure* in SM. C–R relationship was adopted from the literature [9,33].

### 2.2 Control scenarios

The time to reach WHO AQG 2021 was assumed to be different for different control scenarios, corresponding to the three scenarios in this study. Older adults (65+) have a much higher COPD mortality than other age groups. Therefore, in the context of China’s steady aging and population growth, the time required to reach WHO AQG 2021 may affect the ozone control effect [34,35]. Three control scenarios, including aggressive control (SC1), moderate control (SC2), and mild control (SC3), which indicate that WHO AQG 2021 would be reached in 2019 immediately (counterfactual scenario), in 2030, and in 2050, respectively, were considered. Table 1 lists the information for the baseline scenario and the three control scenarios. For different control scenarios, the change in outdoor ozone pollution was considered, as well as the population growth and aging. For the aggressive control scenario, the populations and age distributions were assumed to be the same as in 2019. Under the moderate control scenario, China’s total population will grow to 1415 million by 2030 [35]. With the urbanization increasing to 70% [36], the urban population obtained under SC2 is 990.5 million. By 2050, China’s urbanization was predicted to be 75% [37], and the population will decrease to 1294 million [35]. Therefore, the urban population obtained under mild control scenario is 970.5 million. The projected aging rates in 2030 and 2050 are 18.9% and 29.1%, respectively [35].

**Table 1:** Information of different estimation scenarios.

Scenarios	Year reaching WHO AQG 2021	Highest seasonal outdoor ozone concentration (μg/m^3^)	Population in urban China (million)	Population of aged >65 years old (million, percentage)
2019 (baseline scenario)	–	Mean (standard deviation) 115.2 (22.5)	857.2	98.6 (11.5%)
SC1 (aggressive control)	2019	60 (WHO AQG 2021)	857.2	98.6 (11.5%)
SC2 (moderate control)	2030	60 (WHO AQG 2021)	990.5	187.2 (18.9%)
SC3 (mild control)	2050	60 (WHO AQG 2021)	970.5	282.4 (29.1%)

### 2.3 Analytical model of source-specific exposure concentrations

We used a source-specific exposure model to determine the ozone exposure from indoor and outdoor sources. At different times of a day, indoor ozone could be originated from the emission of indoor ozone-emitting devices and migration of ambient air. Seven indoor ozone-emitting devices were considered in this study, including ozone disinfection machines, ozone disinfection cupboards, copiers, laser printers, fruit/vegetable sterilizers, shoe sanitizers, and ozone-emitting air purifiers (such as ionization purifiers). The indoor ozone concentrations from indoor and outdoor sources were expressed as follows:

(1) $$\begin{array}{c} \frac{{dC}_{i,i,t}}{dt}=\frac{\sum_{j}S_{j,t}}{V}-\left(a_{t}+k\right)C_{i,i,t} \end{array}$$

(2) $$\frac{{dC}_{i,o,t}}{dt}=a_{t}PC_{o,t}-\left(a_{t}+k\right)C_{i,o,t}$$

(3) $$\begin{array}{c} C_{i,t}=C_{i,i,t}+C_{i,o,t} \end{array}$$

where subscript *t* represents the time of day; *C*_*i,t*_ and *C*_*o,t*_ are indoor and outdoor ozone concentrations, μg/m^3^; *C*_*i,i,t*_ and *C*_*i,o,t*_ are indoor ozone concentrations from indoor and outdoor sources, respectively, μg/m^3^; *a*_*t*_ is the air change rate, /h; *S*_*j,t*_ is the emission rate of ozone from source *j*, μg/h; *k* is the total surface removal rate of ozone indoors, /h; *P* is the penetration factor of ozone, the value of which was considered as 1; *V* is the room volume, m^3^.

The gender-, age-, and city-specific ozone exposure concentration could be calculated as follows:

(4) $$C_{e,t}=C_{i,t}\alpha_{i,t}+C_{o,t}\alpha_{o,t}=C_{i,i,t}\alpha_{i,t}+C_{i,o,t}\alpha_{i,t}+C_{o,t}\alpha_{o,t}$$

where *C*_*e,t*_ is the exposure concentration of ozone, μg/m^3^; *α*_*i,t*_ and *α*_*o,t*_ represent the environment a person is in, with values of 1 and 0, respectively, an indoor environment and the opposite in an outdoor environment. *C*_*i,i,t*_*α*_*i,t*_ and (*C*_*i,o,t*_*α*_*i,t*_ + *C*_*o,t*_*α*_*o,t*_) represent exposure concentration from indoor and outdoor sources, respectively.

People have different activity intensities when they are indoors and outdoors, and high-intensity activities are usually performed outdoors. Different activity intensities indicate different breathing rates. Considering that the main pathway of ozone exposure is inhalation, we adjusted ozone exposure by breathing rates in different micro-environments. Three typical activity patterns for different breathing rates, including sitting quietly, sleeping indoors, and slightly exercising outdoors, were considered to represent the main types of activity intensity in daily life [23]. The breathing rates adjusted exposure concentration (*C*_*BRAe*_) of ozone could be expressed as follows:

(5) $$\begin{array}{c} C_{BRAe}\left(t_{1},t_{2}\right)=\frac{\int_{t_{1}}^{t_{2}}C_{e,t}Q_{t}dt}{\int_{t_{1}}^{t_{2}}Q_{t}dt} \end{array}$$

where *Q*_*t*_ is the breath rate for the activity.

### 2.4 Analytical model of source-specific COPD deaths

C–R relationship has been used to describe the mortality risks related to long-term ozone exposure, which was represented by the highest seasonal (six-month) daily maximum of 8-h average (DMA8) [9]. The RR could be calculated by the following [9]:

(6) $$\begin{array}{c} RR=\left\{ \begin{array}{l} 1\;for\;C_{o,HS}\leq LCC\\ \exp[\beta \left(C_{o,HS}-LCC\right)]\;\;for\;C_{o,HS}>LCC \end{array}\right. \end{array}$$

where *C*_*o,HS*_ is the highest seasonal DMA8 outdoor ozone concentration, ppb; LCC is the low concentration cutoff below which the effect estimate (*β*) has not been evaluated for COPD, which is uniformly distributed from 29.1 to 35.7 ppb [9]; *β* is the effect estimate, which could be calculated from the hazard ratio with 10 ppb increase of ozone concentration; the hazard ratio for COPD was 1.06 [9,33]*.* As RR should be associated with the exposure concentration, instead of outdoor concentration, we calculated the highest seasonal exposure equivalent ambient ozone concentration (*C*_*o,e,HS*_) and used it to replace *C*_*o,HS*_ in Eq. 6 [22,38]:

(7) $$\begin{array}{c} C_{o,e,HS}={\left(\frac{C_{BRAe,DMA8}}{f_{\exp}}\right)}_{HS} \end{array}$$

where *C*_*BRAe,DMA8*_ is the DMA8 breathing rates adjusted exposure concentration; *f*_*exp*_ is the exposure factor of ozone (defined as the ratio of exposure from outdoor ozone sources and outdoor concentrations) for the season with the highest DMA8 exposure concentration. Therefore, the RRs, population attributable fraction (PAF) of the baseline scenario, and potential impact fractions (PIFs) of ozone control scenarios for COPD were calculated by the following:

(8) $$\begin{array}{c} RR_{k}=\left\{ \begin{array}{l} 1\;for\;C_{o,e,HS,k}\leq LCC\\ \exp\left[\beta \left(C_{o,e,HS,k}-LCC\right)\right]\;for\;C_{o,e,HS,k}>LCC \end{array}\right. \end{array}$$

(9) $$\begin{array}{c} PAF=\frac{RR_{0}-1}{RR_{0}}\;for\;k=0 \end{array}$$

(10) $$\begin{array}{c} PIF_{k}=\frac{RR_{k}-RR_{0}}{RR_{k}}\;\;\;\;\;\;\;\;\;\;\;\;\;\;for\;k=1,2,3 \end{array}$$

where *k* represents the four estimation scenarios, with value of 0–3, indicating the baseline scenario (*k* = 0) and three control scenarios (*k* = 1, 2, 3, see Fig. 1), respectively.

The deaths for COPD (gender-, age-, and city-specific) attributable to ozone under different scenarios (*Death*_*k*_) were calculated as follows [9]:

(11) $$\begin{array}{c} Death_{k}=\left\{\begin{array}{ll} PAF\times Mort\times Pop_{k} & for\;k=0\\ Death_{0}-Death_{R,k} & for\;k=1,2,3 \end{array}\right) \end{array}$$

(12) $$\begin{array}{c} Death_{R,k}=PIF\times Mort\times Pop_{k}\;\;\;\;\;\;for\;k=1,2,3\; \end{array}$$

where *Mort* is the baseline death rate of COPD of a specific gender, age group, and city; *Pop*_*k*_ is the population of the specific gender, age group, and city under scenarios *k*; *Death*_*R,k*_ is the avoidable death under three control scenarios. The deaths of COPD attributable to indoor (*Death*_*i,k*_) and outdoor (*Death*_*o,k*_) ozone sources of each gender, age group, and city under different scenarios could be expressed by the following [9]:

(13) $$\begin{array}{c} Death_{i,k}=\frac{\left(C_{o,e,HS,k}-C_{o,HS,k}\right)p_{in}}{\left(C_{o,e,HS,k}-C_{o,HS,k}\right)p_{in}+C_{o,HS,k}}\times Death_{k} \end{array}$$

(14) $$\begin{array}{c} Death_{o,k}=\frac{C_{o,HS,k}}{\left(C_{o,e,HS,k}-C_{o,HS,k}\right)p_{in}+C_{o,HS,k}}\times Death_{k}\; \end{array}$$

where *p*_*in*_ is the retention of ozone-emitting devices.

### 2.5 Uncertainty analysis

A Monte Carlo simulation was conducted to obtain the distribution of the exposure concentration of ozone and its correlated COPD deaths. Firstly, we estimated the exposure concentration and the equivalent ambient concentration of ozone with 1000 calculations and obtained the distribution of the highest seasonal equivalent ambient concentration of ozone. When estimating the exposure concentration of ozone, populations with different genders in different cities were considered and divided into 10 age groups: 0–6 months, 7 months–2 years old, 2–4, 5–6, 7–12, 13–18, 19–45, 46–60, 61–80, and >80 years old [23]. The model had been validated by careful comparison and good coincidence between results of simulations and measurements in existing references (section *validation of the ozone exposure estimation model*, Fig. S1). Secondly, the distribution of RRs, PAFs, and PIFs for 10 age groups with 1000 calculations were estimated based on the obtained distribution of the highest seasonal equivalent ambient concentration of ozone. Considering that the baseline death rates were given in 19 age groups, interpolation was used to change PAFs and PIFs into 19 age groups. Lastly, the distribution of age-, gender-, city-, and source-specific COPD deaths for different scenarios was determined. The uncertainty intervals (UIs) of the results were also obtained.

## 3 Results

### 3.1 Ozone concentration in 2019

**Fig. 2:**
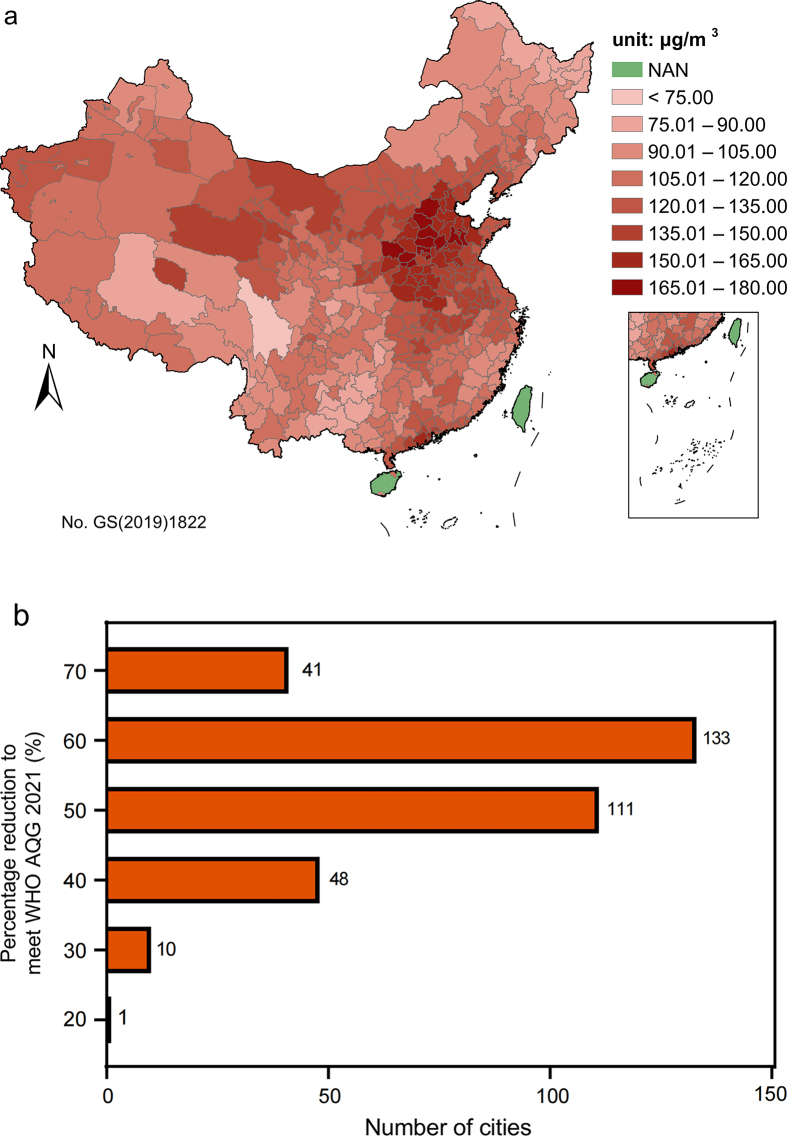
(a) Spatial distribution of the highest seasonal DMA8 of the equivalent ambient ozone concentration in 344 Chinese urban cities in 2019; (b) the number of cities with different levels of ozone reduction to meet WHO AQG 2021. NAN indicates missing data.

The spatial distribution of the highest seasonal DMA8 of the equivalent ambient ozone concentration (*C*_*o,e,HS*_ calculated with Eq. 7) in 344 Chinese cities in 2019 is shown in Fig. 2a. All cities had higher *C*_*o,e,HS*_ in 2019 than WHO AQG 2021, ranging from 70.4 (Ganzi Prefecture) to 173.5 μg/m^3^ (Handan), with the population-weighted average (PWA) concentration of 128.5 μg/m^3^, which is 1.14-fold higher than the WHO AQG 2021 level (60 μg/m^3^). Beijing–Tianjin–Hebei region, central and east China, had more severe ozone pollution than the rest of China. Specifically, 285 cities (83%, 285/344) should reduce their production of ozone (quantified by the highest seasonal DMA8 of the equivalent ambient ozone concentration) by more than 50% to meet WHO AQG 2021, among which amplitude reduction of 41 cities (12%, 41/344) should exceed 70% (Fig. 2b).

### 3.2 COPD deaths attributable to ozone under different scenarios

**Fig. 3:**
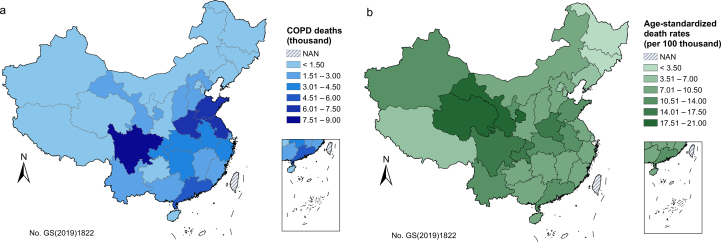
COPD (a) deaths and (b) age-standardized death rates attributable to ozone in all provinces of China in 2019 (baseline scenario). NAN indicates missing data.

Province-specific COPD deaths and age-standardized death rates attributable to ozone of urban China are shown in Fig. 3. COPD deaths attributable to ozone ranged from 20 (Tibet) to 7656 (Sichuan). Among them, Sichuan (7656), Shandong (7196), Henan (6725), Jiangsu (6288), and Guangdong (5612) were the top five provinces with higher ozone-attributable COPD deaths, which might be associated with their higher population density. Generally, high premature COPD deaths were in accordance with high equivalent ambient ozone concentration in Shandong (population weighted average [PWA] *C*_*o,e,HS*_ = 151.7 μg/m^3^) and Henan (PWA *C*_*o,e,HS*_ = 157.2 μg/m^3^), while the smallest COPD deaths might result from the low ozone concentration (PWA *C*_*o,e,HS*_ = 104 μg/m^3^), small population (1.10 million), as well as the low standard population mortalities (30.9 and 38.9 per 100 thousand for male and female, respectively). The five provinces with lowest ozone-attributable COPD deaths were Tibet (20), Ningxia (246), Qinghai (309), Jilin (507), and Heilongjiang (633).

Under SC1, the provinces with highest and lowest ozone-attributable COPD deaths were as follows: Jilin (51), Hunan (45), Liaoning (44), Henan (41), and Shandong (36), as well as Tianjin (<1), Shanghai (<1), Beijing (1), Chongqing (2), and Ningxia (2), respectively. Under SC2, the provinces with highest and lowest ozone-attributable COPD deaths were as follows: Heilongjiang (104), Hunan (99), Shandong (73), Anhui (56), and Hubei (54), as well as Chongqing (<1), Ningxia (1), Qinghai (2), Shanghai (2), and Tianjin (2), respectively. Under SC3, the provinces with highest and lowest ozone-attributable COPD deaths were as follows: Hunan (151), Heilongjiang (145), Shandong (105), Anhui (90), and Sichuan (78), as well as Chongqing (1), Ningxia (3), Qinghai (3), Shanghai (4), and Tianjin (4), respectively. The province-, age-, and gender-specific ozone-attributable COPD deaths under four scenarios are shown in Tables S12–15.

When standardized with age, Heilongjiang and Qinghai had the lowest (3 per 100 thousand) and highest (19 per 100 thousand) age-standardized COPD death rates attributable to ozone, respectively (Fig. 3b). The top five provinces with higher age-standardized COPD death rates were Qinghai, Gansu, Sichuan, Henan, and Chongqing. The lowest age-standardized death rates in Heilongjiang might result from its low ozone concentration (PWA *C*_*o,e,HS*_ = 92.9 μg/m^3^) and low baseline death rates (30.1 and 29.2 per 100 thousand for female and male, respectively). On contrary, Qinghai had high baseline death rates of COPD (99.6 and 90.7 per 100 thousand for female and male, respectively) and ozone concentration (PWA *C*_*o,e,HS*_ = 123.6 μg/m^3^) following Shandong and Henan.

**Fig. 4:**
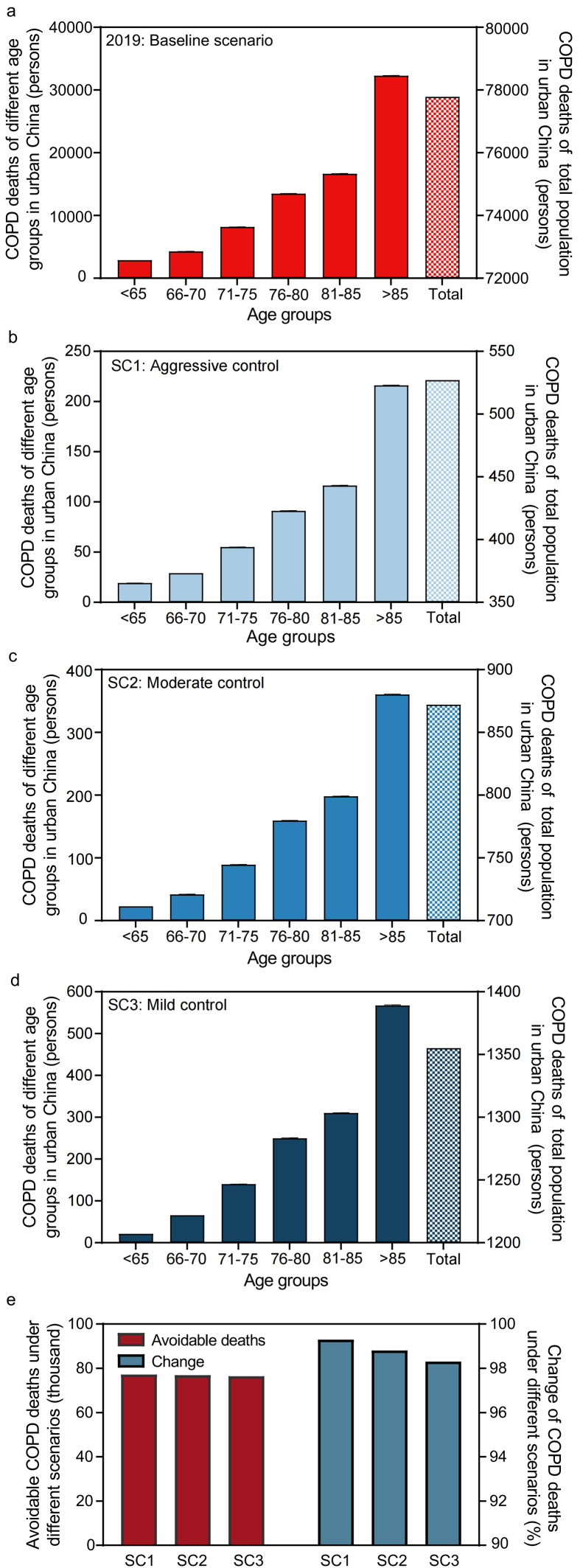
(a)–(d) COPD deaths in 2019 and under three control scenarios; (e) the avoidable deaths for three control scenarios and its contribution to the total deaths in 2019.

Intervention by different levels of control strategies should make profound improvement regarding COPD mortality. Fig. 4 shows the COPD deaths in 2019 (baseline scenario) and under the three ozone control scenarios for different age groups. Without intervention, total premature COPD deaths attributable to ozone in urban China were 77,737 (95% UI 77,608–77,865) in 2019. Older adults over 65 and 80 years old accounted for 96.3% and 62.9% of the total deaths, respectively. Control strategy toward ozone management is predicted to protect the older against COPD. Under SC1 scenario, an estimated 527 (95% UI 510–562) people will die of COPD, of which 508 (96.4%) are older than 65 years old. In other words, 77,210 deaths of COPD would be avoided compared to 2019, accounting for 99.3% of the total COPD deaths attributable to ozone in 2019 (Fig. 4e). The difference in COPD deaths between the baseline scenario and SC1 only resulted from decreased outdoor ozone concentration. With a moderate control strategy, there will be 872 (95% UI 829–915) COPD deaths attributable to ozone in 2030, of which the elderly contributes 97.4% (849). Compared to the baseline scenario in 2019, 76,865 deaths would be avoided, accounting for 98.9% of total COPD deaths in 2019. Due to population and urbanization growth from 2019 to 2030, COPD deaths due to ozone under SC2 were higher than those under SC1. If a mild control strategy is adopted to meet WHO AQG 2021 in 2050, COPD deaths attributable to ozone would be 1355 (95% UI 1287–1422), of which 1333 are over 65 years old. With the mild control of outdoor ozone, an estimated 76,382 deaths of COPD would be avoided, accounting for 98.3% of the total COPD deaths of the baseline scenario in 2019.

### 3.3 Contribution of outdoor and indoor ozone sources to COPD deaths

**Fig. 5:**
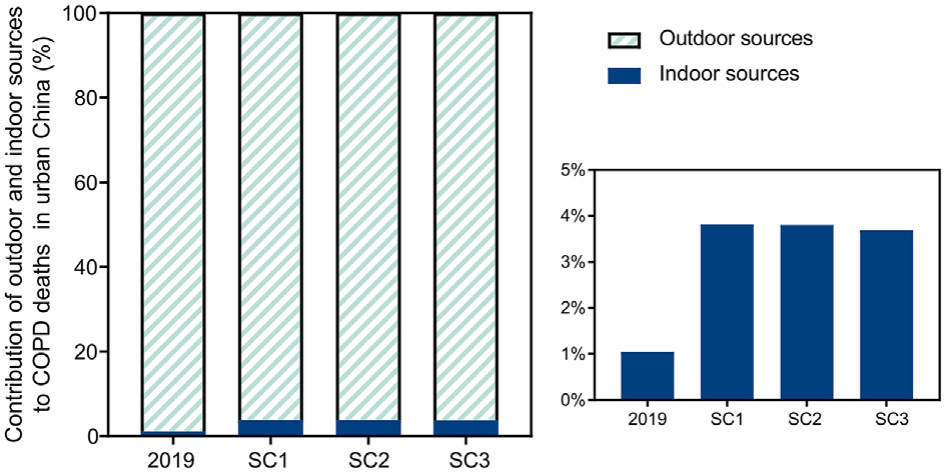
Contributions of outdoor and indoor ozone sources to the ozone-attributable COPD deaths in 2019 and three control scenarios. SC1, aggressive control; SC2, moderate control; SC3, mild control.

The contribution of outdoor and indoor sources of ozone to COPD deaths varied widely (Fig. 5.). In 2019, indoor ozone sources contributed about 1.1% (812 persons) to the total ozone-attributable COPD deaths. With the decrease of outdoor ozone concentration by different control strategies, the contributions of indoor ozone sources increase to 3.8% (20 persons), 3.8% (33 persons), and 3.7% (50 persons) under SC1, SC2, and SC3, respectively.

## 4 Discussions

In this study, ozone-attributable COPD deaths were estimated in 2019 and when outdoor ozone concentration reduced to WHO AQG 2021 in 2019, 2030, and 2050, respectively. COPD deaths attributable to ozone were 77,737 in 2019, and 527, 872, 1355 if the outdoor ozone concentration is reduced to the WHO AQG 2021 level in 2019 (counterfactual scenario), 2030, and 2050, respectively, in urban China. Indoor ozone sources contribute to less than 5% of ozone-attributable COPD deaths.

In previous studies, ozone-attributable COPD deaths have been investigated in different years in China. The study concerning 2014 had the estimation of 89.4 thousand of COPD deaths attributable to ozone in China [39]. An estimation of 46 thousand ozone-attributable premature COPD deaths was reported based on machine learning emulation in 2015 in China [40]. Yin et al. [41] reported a death of 178.2 thousand for COPD attributable to ozone across China in 2017. Xiao et al. [42] estimated the premature COPD deaths from 2013 to 2020, of which the values were about 99, 97, 97, 112, 131, 134, 166, and 148 thousand, respectively. The estimated premature COPD deaths attributable to ozone in this study in urban China accounted for 46.8% of that estimated in the literature in 2019 in China (including both rural and urban areas) [42], indicating that ozone-attributable mortality burden of COPD in rural China was greater than that in urban areas. Differences appeared between studies focusing on the same year. Except for the study of Lin et al. [39], which used the running three-month maximum of DMA1 ozone concentration and a LCC of 37.6 ppb to estimate the RR, other studies mentioned above used the same C–R curve and LCC to that used in this study and highest seasons DMA8 ozone concentrations. However, the methods used to determine ozone concentrations, population sizes, and baseline death rates varied across studies, which may explain differences in ozone-attributable COPD deaths in the same year between different studies.

The spatial distribution of ozone-attributable COPD deaths was determined by the population density, ozone concentration, and the baseline death rates. Sichuan had the highest COPD deaths, followed by Shandong and Henan. In 2014 and 2017, the deaths of COPD in Sichuan also ranked first in China [39,41,43], and in 2015, it ranked fifth in China [44], and the main explanation of which was the high density of population coupled with the relatively high ozone concentration in the Sichuan-Chongqing area. Sichuan, Henan, and Shandong were among the five provinces with the highest population density in China. However, the baseline death rate of COPD was quite high in Sichuan, which ranked third and fourth of all provinces in 2019 for male and female, respectively. What is more, the age-standardized baseline death rates of males in Sichuan were 2.5 and 2.8 times of that in Henan and Shandong, while the age-standardized baseline death rates of females were 2.2 times and 2.5 times of that in Henan and Shandong. High baseline death rates explained why Sichuan had lower ozone concentration, a comparable population but higher COPD deaths than Henan and Shandong. Even though the ozone concentration was relatively low in this study, Sichuan still had the highest COPD deaths and deaths rate across China. Therefore, the prevention and treatment of COPD should be paid attention to in Sichuan. Regular pulmonary function monitoring and other means should be carried out to detect COPD as early as possible and intervene promptly.

PM_2.5_ is another environmental risk factor for COPD. In 2017, both ambient PM_2.5_ (851.7 thousand) and household air pollution from solid fuels (271.1 thousand) contributed to more COPD deaths than that of ozone (178.2 thousand) in China [41]. However, thanks to the proper control strategy adopted in China, the sources of indoor PM_2.5_ have been strictly monitored, and the concentration of outdoor PM_2.5_ has also dropped significantly (from 118 μg/m^3^ in 2013 to 33 μg/m^3^ in 2020) [45], which should directly result in a decrease in COPD mortality attributable to both indoor and outdoor PM_2.5_. Contrary to the improvement of PM_2.5_ pollution, ozone pollution is becoming pervasive both indoors and outdoors. As for indoor emitted ozone, its attributable COPD deaths appeared to be negligible in urban China. The average (± standard deviation, SD) retention rate of indoor ozone-emitting devices was 0.68 (± 0.10), with a range of 0.43–0.88. However, the usage time of ozone-emitting devices was short in residences. The ozone disinfection cupboard was the device with the longest usage time of all the six considered indoor-emitting source devices with an average (± SD) usage time of only 17 (± 23) mins. Since indoor emissions of ozone remained unchanged, the influence of increased usage of indoor ozone-emitting devices could not be reflected in the current study. At the same time, the use of potential ozone-emitting devices, such as ultraviolet disinfecting lamps and ozone generators, has become more prevalent, perhaps due to the COVID-19 pandemic since 2020 and sanitation initiatives [46,47]. Indoor sources of ozone may become a blind spot in public health due to insufficient measurements and remote sensing techniques.

Since the launch of Air Pollution Prevention and Control Action Plan (APPCAP) in 2013, among the six criteria pollutants (ozone, PM_2.5_, inhalable particulate matter, carbon monoxide, nitrogen dioxide, and sulfur dioxide), ozone is the only air pollutant with an increasing outdoor concentration [45,48], which requires urgent development of effective control strategies. In particular, the results of this study revealed that the gap between outdoor ozone concentration in 2019 and WHO AQG 2021 was still large, and the population aging offset the health benefits of outdoor ozone control. Our results (Fig. 4) show that COPD deaths under the mild control scenario were 2.6 and 1.6 times that under aggressive and moderate control, respectively. The difference mainly resulted from the population aging in urban China. The population of urban China would decrease by 2% from 2030 to 2050, approximately 20 million people. However, the elderly (65+) population in 2050 would reach 282.4 million, which is 51% (95.2 million) and 186% (183.8 million) higher than that in 2030 and 2019 in urban China. Projections of mortality burden of respiratory disease attributable to ozone in 2100 under different SSPs and in 2050 under different RCPs also report the offsetting health benefits of lower ozone concentrations from population aging [19,21]. Therefore, stricter ozone control measures should be taken to reduce the concentration to a safe level as soon as possible.

Ozone is mainly formed from the photochemical reactions of nitrogen oxides (NOx) and volatile organic compounds (VOCs) in the atmosphere. Restricting the emission of NOx and VOCs is an important means of controlling the formation of ozone. Control strategies of NOx have been conducted to restrict ozone in China for a couple of years. However, controlling only one precursor has made little difference in the ozone concentration. Therefore, a synergistic emission reduction of both NOx and VOCs should be performed, which has also been considered in the *Fourteenth Five-Year Plan* in China. Further adjustment of energy structure would be an effective measure that also helps to reduce carbon emissions. Traditional fossil energy vehicles could be replaced with electric and hybrid vehicles. Governments could strengthen the construction and development of public transportation to reduce the frequency of private vehicles as well as vehicle exhaust emissions. COPD prevalence is an epidemic in China, and various public health efforts are aimed at increasing awareness and treatment. If ozone concentrations are not controlled as a prevention measure, these efforts may be futile.

One limitation of this study is that the C–R relationship and the LCCs were from GBD 2019, which was determined based on the global population. Different populations may have different vulnerabilities to ozone, leading to different C–R relationships and LCCs. Acclimation may also be a factor. Therefore, there might be a bias in our estimation of COPD deaths attributable to ozone. However, the current used C–R curves and LCCs reported in GBD 2019 may be reasonable as the GBD 2019 took into account the global population, including the Chinese. Future studies should quantify the association between long-term ozone exposure and mortality burden of COPD for China. Another limitation may be that we did not incorporate other diseases associated with ozone, such as cardiovascular disease [49], whose burden and mortality should be estimated by future studies.

## 5 Conclusions and implications

In this study, source-specific exposure concentrations of ozone were simulated in urban China. Based on the concentration-response relationship of long-term ozone exposure and RRs for COPD, the ozone attributable COPD deaths were estimated under the baseline scenario in 2019, and under three ozone control scenarios, when outdoor ozone concentration decreased to WHO AQG 2021 level in 2019, 2030, and 2050, respectively. We obtained the contribution of indoor-emitting ozone to COPD deaths and avoidable deaths of COPD under different ozone control scenarios. To our knowledge, this is the first study of ozone-attributable COPD deaths considering both indoor and outdoor ozone sources and elucidates the gap of ozone concentration that needs to bridge to meet WHO AQG 2021. Studying indoor ozone sources further improves the accuracy of the ozone exposure estimation in China.

Our results indicated that highest seasonal ozone concentration in 2019 was 2.1 times that of WHO AQG 2021, with a gap of 68.5 μg/m^3^ to be bridged. COPD deaths attributed to ozone were 77,737 in 2019, 98% of which could be avoided if the outdoor ozone concentration decreased to WHO AQG 2021 level. The ozone-attributable COPD deaths varied with geographic region. Our findings contribute to a better understanding of the COPD deaths attributable to ozone in urban China, suggesting that indoor ozone contributes little to COPD deaths and reducing outdoor ozone to WHO AQG level is effective in reducing ozone-attributable COPD deaths. Also, population growth and aging influence the effect of ozone control. These findings further advocate reducing outdoor ozone concentration to improve respiratory health and reveal the urgency of outdoor ozone measures. Gaseous air pollutants may not respond to particulate matter pollution control measures, due to different emission sources. Even under the context of low air pollution exposure, other prevention measures, early detection, and treatment of COPD can improve prognosis.

## Author contributions

MY Yao designed the study and planned the analysis, performed the model analysis, analyzed the results, interpreted the results, validated and completed all figures, and drafted the manuscript. Y Hu collected data. A Zhang collected data. JS Ji drafted and commented on the manuscript. B Zhao coordinated and supervised the project, designed the study and planned the analysis, analyzed the results, interpreted the results, and drafted the manuscript. All authors reviewed and approved the final report.
