# Supplementary material for: COPD deaths attributable to ozone in 2019 and future projections using the WHO AQG 2021 in urban China
Source: Eco Environ Health. 2022 Nov 28;1(4):251–8. doi: 10.1016/j.eehl.2022.11.002 (PMC10702908; doi:10.1016/j.eehl.2022.11.002)
Supplement: Multimedia component 1 [file mmc1.docx]

**Supplementary material for “COPD deaths attributable to ozone in 2019 and future projections using the WHO AQG 2021** **in urban China”**

Mingyao Yao^1^, Ying Hu^1^, Ao Zhang^1^, John S. Ji^2^, Bin Zhao^*,1,3^

^1^ Department of Building Science, School of Architecture, Tsinghua University, Beijing, 100084, China.

^2^Vanke School of Public Health, Tsinghua University, Beijing, 100084, China.

^3^ Beijing Key Laboratory of Indoor Air Quality Evaluation and Control, Tsinghua University, Beijing, 100084, China.

**Address for correspondence:**

Bin Zhao, Department of Building Science, School of Architecture, Tsinghua University, Beijing 100084, China. Tel.: +86-10-6277-9995, Fax: +86-10-6277-3461, Email: binzhao@tsinghua.edu.cn.

**Table of Contents**

Contents

[1. Input parameters 3](#_Toc117630421)

[2. Validation of the ozone exposure estimation model. 3](#_Toc117630422)

[Table S1 The retention rates of different ozone-emitting devices of different provinces. 5](#_Toc117630423)

[Table S2 Emission rate distributions of different ozone-emitting devices indoors (Shen 2018). 5](#_Toc117630424)

[Table S3 Window opening probabilities of different times for different seasons. 6](#_Toc117630425)

[Table S4 Probabilities of turning on ozone-emitting devices for different times of day and the length of time with the devices on. 6](#_Toc117630426)

[Table S5 Length of time (mean ± SD) the air purifiers were on for different AQIs. 7](#_Toc117630427)

[Table S6 Baseline death rates of males for different age groups in different provinces. 8](#_Toc117630428)

[Table S7 Baseline death rates of females for different age groups in different provinces. 9](#_Toc117630429)

[Table S8 Proportion for populations with different ages for male in different provinces and municipalities. (Values for a province are used for all the cities in that province.) 11](#_Toc117630430)

[Table S9 Proportion for populations with different ages for females in different provinces and municipalities. (Values for a province are used for all the cities in that province.) 12](#_Toc117630431)

[Table S10 Proportion of the populations with different ages in different provinces and municipalities under different scenarios. (Values for a province are used for all the cities in that province.) 14](#_Toc117630432)

[Table S11: Information regarding the model evaluation cases and the parameter settings for the model. 15](#_Toc117630433)

[References 16](#_Toc117630434)

# 1. Input parameters

**Dynamic estimation:** As eq. 1–5 show, a dynamic simulation of ozone exposure concentration was conducted in this study. We set a time step (*dt*) of 0.01 h (6 mins). During each time step, the ozone concentration was assumed to be stable. Therefore, the indoor ozone concentration from indoor and outdoor ozone sources could be calculated as:

$$\begin{aligned} C_{i,i,t}=(\frac{S_{t-1}}{V}-\left( a_{t-1}+k \right)C_{i,i,t-1})dt+C_{i,i,t-1}\# \end{aligned}(S1)$$

$$\begin{aligned} C_{i,o,t}=(a_{t-1}PC_{o,t-1}-\left( a_{t-1}+k \right)C_{i,o,t-1})dt+C_{i,o,t-1}\# \end{aligned}(S2)$$

The indoor ozone concentration was set as 0, and outdoor ozone concentration from the monitor stations was used at the initial time (*t*=0), respectively. To eliminate the influence of the initial concentration, we started the calculation on December 1^st^, 2018.

**Surface removal rates**: The distributions of surface removal rates by building materials/furniture surfaces and human surfaces were calculated as following[1]:

$$\begin{aligned} k_{i}=\frac{v_{d,i}A_{i}}{V}\# \end{aligned}(S3)$$

where *i* represents the type of the surfaces, namely building materials/furniture surfaces and human surfaces; *v_d,i_* is the deposition velocity of ozone onto surface *i,* m/h; *V* is the room volume, m^3^. The deposition velocities of building materials/furniture surfaces and human surfaces were assumed to be log-normal distributions, which were ln(*v_d, building&furniture_*)*~N*(0.01, 0.32^2^), with a range of 0.49–1.75 m/h, and ln(*v_d,human_*)*~**N*(2.2, 0.78^2^), with a range of 3.1–48.7 m/h, respectively[2, 3]. The value of *A_building&furniture_/V* was assumed to be constant, which was 2.5 /m[2]. The room volume (*V*) could be calculated with the product of the floor area (*S*) and the ceiling height (*H*). The ceiling height was set as 2.6 m[1]. The per capita floor area and the human surface area for different ages, the percentages of the number of occupants in a family, and the human surface areas of all provinces in mainland China were taken from the literature (Table S4 and S6 in Yao, et al. [1]).

**Time schedules of different activities:** As the dynamic estimation was considered, the activity patterns of window opening/closing, ozone-emitting devices turning on/off, and going out/bed/sitting quietly indoors were required. The periods of all the activities in a day were determined by two parameters, the start time of the activity and the duration of the activity. Taking the period of a window opening as an example, we determined the duration of windows open for a day by obtaining the time when people opened their windows (**Table S3**) and the length of time they kept their windows open for a day. For the activity of turning on ozone-emitting devices, the probabilities of turning on ozone-emitting devices (excluding ozone-emitting air purifiers) for different times of day and the length of time with the devices on are shown in **Table S4.** The turning-on of ozone-emitting air purifiers was determined by the outdoor air quality, which was represented by Air Quality Index (AQI). The probabilities of turning on air purifiers of different AQIs could be found in Table S10 in Hu and Zhao[4]. The length of time the air purifiers were on was obtained from our survey, which surveyed 1258 families in 31 provinces in mainland China, sees **Table S5**. The time of sleep was assumed to be uniformly distributed between 21:00 to 24:00 and the duration was set as 8 hours. Time spent outdoors each day is considered continuous, the daily proportion of which was assumed to be log-normal distribution, details see Table 1 and Table S8–S10 in [1]. According a previous survey [5], the mean (±SD) values of time people went outside were 8:00 (±52.2 min), with a range of 6:30–10:00, and 9:48 (±112.2 min), with a range of 8:00–13:00, in weekdays and weekends, respectively. Therefore, the activity for the rest of the day was sitting quietly.

**Age structures:** The Seventh Census gave the province-specific proportions of those younger than 15, 15–65, and older than 65 years old for different genders, respectively. The Six Census gave the proportions of the population every 5 years from 0 to 100 years old for different genders[6]. The interpolation method was used to determine the proportion of the populations of the target 19 age groups in this study. Therefore, the age distributions for males and females were obtained in 2019 and are shown in **Table S8 and S9**, respectively. There is a prediction of the aging rates of 18.9% and 29.1% in 2030 and 2050, respectively[7]. The proportions of those younger than 15 years old in different provinces in 2030 and 2050 were obtained from literature[8]. **Table S9** shows the province-specific proportions of populations younger than 15, 15–65, and older than 65 years old under different scenarios. Similar to 2019, the age distributions for males and females were determined based on the interpolation method in 2030 and 2050.

# 2. Validation of the ozone exposure estimation model.

We used our model to estimate personal concentrations (exposure concentrations) in the four cases investigated in field measurements to evaluate the model. To match the conditions of these studies, the exposure concentrations for populations of similar ages during the same time of year and at the same locations were modeled. **Table S11** shows the information about the field measurements and the corresponding modeling. The results are shown in Figure S1. For Case 1, the personal concentrations of college students in Shanghai were monitored during summertime. The median measured value of the field study was 19.2 ppb with a standard deviation of 13.1 ppb[9], compared to our calculated median value of 20.0 ppb with a standard deviation of 6.7 ppb. For Case 2 (Nanjing), the median measured value in the field study was 12.0 with a standard deviation of 8.3 ppb[10], while our calculated median value was 9.9 with a standard deviation of 4.1 ppb. For Case 3, the median measured value in the field study was 7.0 with a standard deviation of 2.3 ppb [11], while our calculated median value was 10.3 with a standard deviation of 3.8 ppb. For Case 3, the median measured value in the field study was 10 with a mean value of 11 ppb[12], while our calculated median value was 13.8 with a standard deviation of 14.1 ppb. Hence, modeled results for the medians are in reasonable agreement with the measured values, providing some reassurance in terms of the performance of the model.


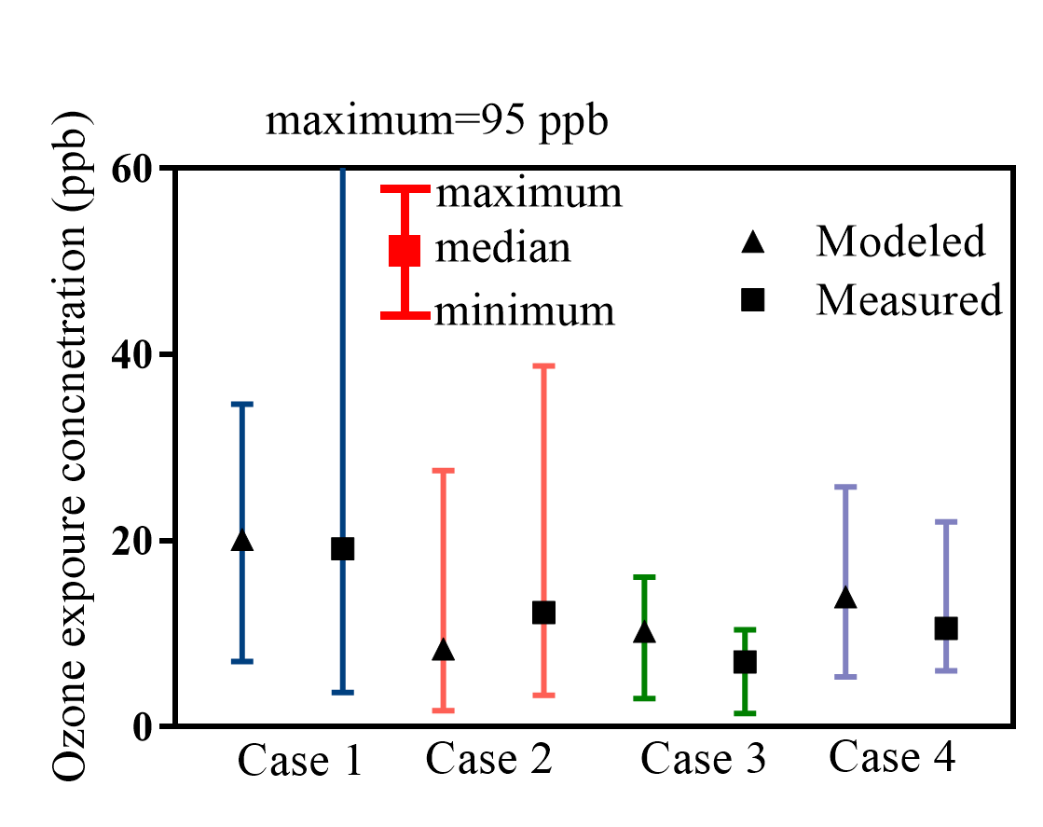


**Figure S1: Validation of the model.**

# Table S1 The retention rates of different ozone-emitting devices of different provinces.

| **Ozone-emitting devices** | **Disinfection machines** | **Ozone disinfection cupboard** | **Laser printers** | **Copiers** | **Fruit/vegetable sterilizers** | **Shoe sanitizers** | **Ozone-emitting air purifiers** |
| --- | --- | --- | --- | --- | --- | --- | --- |
| Beijing | 0.44 | 0.44 | 0.17 | 0.36 | 0.44 | 0.33 | 0.57 |
| Tianjin | 0.36 | 0.54 | 0.39 | 0.32 | 0.29 | 0.32 | 0.58 |
| Hebei | 0.48 | 0.55 | 0.42 | 0.29 | 0.58 | 0.48 | 0.41 |
| Shanxi | 0.48 | 0.58 | 0.52 | 0.52 | 0.52 | 0.48 | 0.44 |
| InnerMongolia | 0.37 | 0.51 | 0.37 | 0.31 | 0.46 | 0.37 | 0.43 |
| Liaoning | 0.31 | 0.41 | 0.22 | 0.19 | 0.50 | 0.25 | 0.43 |
| Jilin | 0.43 | 0.54 | 0.39 | 0.32 | 0.50 | 0.46 | 0.41 |
| Heilongjiang | 0.50 | 0.54 | 0.46 | 0.32 | 0.57 | 0.39 | 0.56 |
| Shanghai | 0.24 | 0.41 | 0.30 | 0.24 | 0.38 | 0.16 | 0.24 |
| Jiangsu | 0.45 | 0.61 | 0.30 | 0.21 | 0.52 | 0.45 | 0.49 |
| Zhejiang | 0.31 | 0.56 | 0.34 | 0.19 | 0.34 | 0.22 | 0.36 |
| Anhui | 0.41 | 0.56 | 0.41 | 0.26 | 0.41 | 0.48 | 0.44 |
| Fujian | 0.30 | 0.57 | 0.37 | 0.27 | 0.27 | 0.20 | 0.42 |
| Jiangxi | 0.33 | 0.47 | 0.40 | 0.30 | 0.30 | 0.30 | 0.44 |
| Shandong | 0.50 | 0.59 | 0.38 | 0.34 | 0.66 | 0.41 | 0.51 |
| Henan | 0.55 | 0.70 | 0.48 | 0.45 | 0.58 | 0.45 | 0.50 |
| Hubei | 0.37 | 0.43 | 0.37 | 0.27 | 0.43 | 0.40 | 0.38 |
| Hunan | 0.46 | 0.61 | 0.29 | 0.25 | 0.43 | 0.39 | 0.34 |
| Guangdong | 0.43 | 0.70 | 0.37 | 0.30 | 0.57 | 0.50 | 0.54 |
| Guangxi | 0.50 | 0.58 | 0.33 | 0.13 | 0.21 | 0.21 | 0.32 |
| Hainan | 0.20 | 0.33 | 0.27 | 0.20 | 0.17 | 0.23 | 0.41 |
| Chongqing | 0.13 | 0.37 | 0.27 | 0.27 | 0.43 | 0.50 | 0.37 |
| Sichuan | 0.21 | 0.42 | 0.30 | 0.12 | 0.30 | 0.30 | 0.26 |
| Guizhou | 0.30 | 0.37 | 0.30 | 0.13 | 0.33 | 0.37 | 0.31 |
| Yunnan | 0.43 | 0.49 | 0.34 | 0.43 | 0.49 | 0.43 | 0.42 |
| Tibet | 0.23 | 0.23 | 0.35 | 0.32 | 0.19 | 0.13 | 0.28 |
| Shaanxi | 0.53 | 0.50 | 0.31 | 0.31 | 0.44 | 0.41 | 0.61 |
| Gansu | 0.35 | 0.53 | 0.35 | 0.35 | 0.50 | 0.47 | 0.41 |
| Qinghai | 0.39 | 0.45 | 0.26 | 0.29 | 0.39 | 0.35 | 0.29 |
| Ningxia | 0.24 | 0.45 | 0.48 | 0.41 | 0.31 | 0.28 | 0.46 |
| Xinjiang | 0.53 | 0.59 | 0.41 | 0.34 | 0.50 | 0.47 | 0.44 |

# Table S2 Emission rate distributions of different ozone-emitting devices indoors [13].

| **Emission rate (mg/h)** | | **Mean** | **Standard deviation** | **Minimum** | **Maximum** |
| --- | --- | --- | --- | --- | --- |
| Disinfection machines | Log-normal | 3.83 | 1.11 | 5.00 | 300.00 |
| Ozone disinfection cupboard | normal | 0.41 | 0.26 | 0.01 | 1.00 |
| Laser printers | Log-normal | 0.77 | 1.02 | 0.10 | 10.00 |
| Copiers | normal | 0.86 | 0.85 | 0.01 | 4.00 |
| Fruit/vegetable sterilizers | normal | 0.41 | 0.26 | 0.01 | 1.00 |
| Shoe sanitizers | normal | 0.41 | 0.26 | 0.01 | 1.00 |
| Ozone-emitting air purifiers | log-normal | 0.51 | 1.47 | 0.00 | 50.00 |

# Table S3 Window opening probabilities of different times for different seasons.

|  | **Spring** | | **Summer** | | **Fall** | | **Winter** | |
| --- | --- | --- | --- | --- | --- | --- | --- | --- |
|  | **Weekday** | **Weekend** | **Weekday** | **Weekend** | **Weekday** | **Weekend** | **Weekday** | **Weekend** |
| 0:00–1:00 | 0.05 | 0.07 | 0.08 | 0.10 | 0.00 | 0.08 | 0.04 | 0.07 |
| 1:00–2:00 | 0.04 | 0.07 | 0.07 | 0.10 | 0.00 | 0.06 | 0.02 | 0.05 |
| 2:00–3:00 | 0.00 | 0.00 | 0.01 | 0.02 | 0.00 | 0.01 | 0.00 | 0.01 |
| 3:00–4:00 | 0.00 | 0.00 | 0.01 | 0.01 | 0.00 | 0.00 | 0.00 | 0.00 |
| 4:00–5:00 | 0.00 | 0.00 | 0.01 | 0.01 | 0.00 | 0.00 | 0.00 | 0.00 |
| 5:00–6:00 | 0.01 | 0.01 | 0.08 | 0.01 | 0.00 | 0.00 | 0.00 | 0.00 |
| 6:00–7:00 | 0.04 | 0.06 | 0.12 | 0.04 | 0.00 | 0.04 | 0.03 | 0.01 |
| 7:00–8:00 | 0.07 | 0.06 | 0.12 | 0.07 | 0.00 | 0.08 | 0.08 | 0.03 |
| 8:00–9:00 | 0.08 | 0.08 | 0.05 | 0.08 | 0.06 | 0.13 | 0.12 | 0.08 |
| 9:00–10:00 | 0.07 | 0.08 | 0.01 | 0.07 | 0.21 | 0.10 | 0.13 | 0.10 |
| 10:00–11:00 | 0.05 | 0.10 | 0.01 | 0.05 | 0.33 | 0.08 | 0.10 | 0.10 |
| 11:00–12:00 | 0.04 | 0.08 | 0.01 | 0.04 | 0.27 | 0.06 | 0.06 | 0.05 |
| 12:00–13:00 | 0.03 | 0.04 | 0.02 | 0.02 | 0.12 | 0.06 | 0.05 | 0.05 |
| 13:00–14:00 | 0.03 | 0.04 | 0.02 | 0.02 | 0.00 | 0.04 | 0.03 | 0.05 |
| 14:00–15:00 | 0.01 | 0.01 | 0.01 | 0.01 | 0.00 | 0.02 | 0.03 | 0.06 |
| 15:00–16:00 | 0.01 | 0.03 | 0.02 | 0.01 | 0.00 | 0.02 | 0.03 | 0.04 |
| 16:00–17:00 | 0.02 | 0.01 | 0.02 | 0.01 | 0.00 | 0.04 | 0.02 | 0.02 |
| 17:00–18:00 | 0.03 | 0.03 | 0.04 | 0.02 | 0.00 | 0.04 | 0.02 | 0.00 |
| 18:00–19:00 | 0.07 | 0.04 | 0.05 | 0.06 | 0.00 | 0.04 | 0.03 | 0.02 |
| 19:00–20:00 | 0.07 | 0.06 | 0.05 | 0.06 | 0.00 | 0.02 | 0.05 | 0.03 |
| 20:00–21:00 | 0.08 | 0.04 | 0.04 | 0.07 | 0.00 | 0.01 | 0.05 | 0.03 |
| 21:00–22:00 | 0.06 | 0.01 | 0.04 | 0.02 | 0.00 | 0.00 | 0.03 | 0.04 |
| 22:00–23:00 | 0.06 | 0.00 | 0.05 | 0.03 | 0.00 | 0.02 | 0.03 | 0.04 |
| 23:00–24:00 | 0.07 | 0.07 | 0.10 | 0.09 | 0.00 | 0.07 | 0.04 | 0.09 |

# Table S4 Probabilities of turning on ozone-emitting devices for different times of day and the length of time with the devices on.

|  |  |  | **Disinfection machines** | **Ozone disinfection cupboard** | **Laser printers** | **Copiers** | **Fruit/vegetable sterilizers** | **Shoe sanitizers** |
| --- | --- | --- | --- | --- | --- | --- | --- | --- |
| Probabilities of turning on the ozone-emitting devices for different times of day | Weekday | 6:00–12:00 | 0.31 | 0.18 | 0.31 | 0.29 | 0.30 | 0.15 |
|  |  | 12:00–18:00 | 0.16 | 0.35 | 0.41 | 0.37 | 0.42 | 0.20 |
|  |  | 18:00–22:00 | 0.37 | 0.32 | 0.17 | 0.23 | 0.21 | 0.35 |
|  |  | 22:00–24:00 | 0.05 | 0.09 | 0.00 | 0.01 | 0.02 | 0.21 |
|  |  | None | 0.12 | 0.05 | 0.11 | 0.11 | 0.06 | 0.09 |
|  | Weekend | 6:00–12:00 | 0.35 | 0.23 | 0.32 | 0.34 | 0.34 | 0.17 |
|  |  | 12:00–18:00 | 0.26 | 0.40 | 0.38 | 0.38 | 0.44 | 0.30 |
|  |  | 18:00–22:00 | 0.30 | 0.28 | 0.17 | 0.17 | 0.19 | 0.32 |
|  |  | 22:00–24:00 | 0.06 | 0.07 | 0.00 | 0.00 | 0.02 | 0.18 |
|  |  | None | 0.02 | 0.02 | 0.13 | 0.11 | 0.01 | 0.03 |
| Length of time with the devices on | Weekday | Mean±SD (h) | 0.17±0.32 | 0.28±0.38 | 0.11±0.26 | 0.09±0.22 | 0.17±0.28 | 0.14±0.26 |
|  | Weekend | Mean±SD (h) | 0.21±0.35 | 0.28±0.37 | 0.11±0.25 | 0.09±0.22 | 0.22±0.32 | 0.17±0.29 |

# Table S5 Length of time (mean ± SD) the air purifiers were on for different AQIs.

|  | **AQI<50** | **50≤AQI<100** | **100≤AQI<150** | **150≤AQI<200** | **200≤AQI<300** | **AQI≥300** |
| --- | --- | --- | --- | --- | --- | --- |
| Beijing | 5.3±2.1 | 5.3±2.1 | 2.0±0.7 | 4.6±3.0 | 7.2±5.1 | 7.2±5.1 |
| Tianjin | 5.3±2.1 | 5.3±2.1 | 5.7±9.0 | 6.1±8.0 | 9.7±8.6 | 9.7±8.6 |
| Hebei | 5.3±5.8 | 5.3±5.8 | 4.4±6.5 | 6.7±6.2 | 10.5±7.8 | 10.5±7.8 |
| Shanxi | 5.3±2.1 | 5.3±2.1 | 2.5±1.1 | 4.2±2.6 | 8.9±6.1 | 8.9±6.1 |
| Inner Mongolia | 1.0±2.1 | 1.0±2.1 | 3.6±3.2 | 5.0±4.2 | 8.9±8.3 | 8.9±8.3 |
| Liaoning | 2.5±2.1 | 2.5±2.1 | 2.3±1.8 | 4.5±4.6 | 7.8±6.6 | 7.8±6.6 |
| Jilin | 2.5±2.1 | 2.5±2.1 | 2.7±2.4 | 5.5±5.4 | 9.1±6.8 | 9.1±6.8 |
| Heilongjiang | 2.5±2.1 | 2.5±2.1 | 4.4±7.0 | 5.1±5.4 | 8.6±7.5 | 8.6±7.5 |
| Shanghai | 8.0±2.1 | 8.0±2.1 | 4.1±3.0 | 6.7±6.1 | 9.6±6.9 | 9.6±6.9 |
| Jiangsu | 2.5±2.1 | 2.5±2.1 | 4.6±5.5 | 6.0±5.8 | 9.1±7.1 | 9.1±7.1 |
| Zhejiang | 2.0±2.1 | 2.0±2.1 | 3.4±4.0 | 6.2±5.0 | 9.5±6.8 | 9.5±6.8 |
| Anhui | 3.0±2.1 | 3.0±2.1 | 2.6±2.4 | 4.2±2.9 | 7.5±5.7 | 7.5±5.7 |
| Fujian | 2.0±2.1 | 2.0±2.1 | 3.4±3.0 | 5.5±5.0 | 10.4±8.1 | 10.4±8.1 |
| Jiangxi | 1.0±2.1 | 1.0±2.1 | 2.6.0±2.1 | 5.7±5.5 | 10.1±7.9 | 10.1±7.9 |
| Shandong | 1.0±2.1 | 1.0±2.1 | 2.6±2.2 | 4.2±3.7 | 7.1±5.7 | 7.1±5.7 |
| Henan | 5.3±2.1 | 5.3±2.1 | 2.7±2.4 | 4.9±4.2 | 8.5±6.8 | 8.5±6.8 |
| Hubei | 1.0±2.1 | 1.0±2.1 | 2.6±1.4 | 5.0±4.7 | 7.7±6.1 | 7.7±6.1 |
| Hunan | 2.3±0.6 | 2.3±0.6 | 4.2±5.1 | 6.6±5.0 | 12.1±7.9 | 12.1±7.9 |
| Guangdong | 4.8±3.6 | 4.8±3.6 | 3.9±4.4 | 5.8±5.2 | 9.9±7.6 | 9.9±7.6 |
| Guangxi | 2.0±1.4 | 2.0±1.4 | 2.6±1.1 | 5.6±5.5 | 10.9±8.3 | 10.9±8.3 |
| Hainan | 2.0±2.1 | 2.0±2.1 | 2.3±0.5 | 6.6±4.2 | 12.8±9.1 | 12.8±9.1 |
| Chongqing | 0 | 0 | 3.1±2.1 | 4.1±3.2 | 8.0±6.9 | 8.0±6.9 |
| Sichuan | 1.0±2.1 | 1.0±2.1 | 2.8±2.5 | 5.9±5.6 | 9.7±7.8 | 9.7±7.8 |
| Guizhou | 1.0±2.1 | 1.0±2.1 | 3.3±2.5 | 4.6±3.4 | 8.8±7.4 | 8.8±7.4 |
| Yunnan | 2.5±0.7 | 2.5±0.7 | 3.1±1.6 | 5.3±3.4 | 11.3±7.8 | 11.3±7.8 |
| Xizang | 1.0±2.1 | 1.0±2.1 | 2.4±1.6 | 6.0±5.7 | 14.0±14.1 | 14.0±14.1 |
| Shannxi | 5.3±2.1 | 5.3±2.1 | 2.5±1.3 | 5.2±5.0 | 8.9±7.2 | 8.9±7.2 |
| Gansu | 1.0±2.1 | 1.0±2.1 | 2.4±1.5 | 4.5±2.3 | 8.4±4.9 | 8.4±4.9 |
| Qinghai | 1.0±2.1 | 1.0±2.1 | 2.7±2.1 | 7.4±7.8 | 10.2±9.4 | 10.2±9.4 |
| Ningxia | 1.0±2.1 | 1.0±2.1 | 8.0±2.1 | 5.0±3.5 | 10.7±8.7 | 10.7±8.7 |
| Xinjiang | 1.3±0.6 | 1.3±0.6 | 2.4±1.6 | 4.0±2.9 | 7.2±5.7 | 7.2±5.7 |

# Table S6 Baseline death rates of males for different age groups in different provinces.

|  | **0-1** | **1-5** | **5-10** | **10-15** | **15-20** | **20-25** | **25-30** | **30-35** | **35-40** | **40-45** | **45-50** | **50-55** | **55-60** | **60-65** | **65-70** | **70-75** | **75-80** | **80-85** | **>85** |
| --- | --- | --- | --- | --- | --- | --- | --- | --- | --- | --- | --- | --- | --- | --- | --- | --- | --- | --- | --- |
| Beijing | 0.00 | 0.00 | 0.00 | 0.00 | 0.00 | 0.00 | 0.00 | 0.02 | 0.09 | 0.10 | 0.52 | 1.16 | 2.67 | 9.04 | 21.79 | 54.77 | 114.20 | 231.18 | 804.92 |
| Tianjin | 0.00 | 0.00 | 0.00 | 0.00 | 0.00 | 0.00 | 0.00 | 0.03 | 0.11 | 0.11 | 0.58 | 1.30 | 3.00 | 10.15 | 24.46 | 61.48 | 128.17 | 259.47 | 903.41 |
| Hebei | 0.00 | 0.00 | 0.00 | 0.00 | 0.00 | 0.00 | 0.00 | 0.03 | 0.11 | 0.12 | 0.63 | 1.40 | 3.22 | 10.91 | 26.30 | 66.10 | 137.81 | 278.98 | 971.34 |
| Shanxi | 0.00 | 0.00 | 0.00 | 0.00 | 0.00 | 0.07 | 0.00 | 0.00 | 0.18 | 0.53 | 1.35 | 2.91 | 9.84 | 21.80 | 47.23 | 110.16 | 249.23 | 494.93 | 1223.95 |
| Inner Mongolia | 0.00 | 0.00 | 0.00 | 0.00 | 0.00 | 0.11 | 0.00 | 0.00 | 0.29 | 0.83 | 2.12 | 4.56 | 15.43 | 34.19 | 74.09 | 172.81 | 390.98 | 776.43 | 1920.11 |
| Liaoning | 0.00 | 0.00 | 0.00 | 0.00 | 0.00 | 0.00 | 0.00 | 0.03 | 0.13 | 0.13 | 0.70 | 1.56 | 3.59 | 12.17 | 29.33 | 73.73 | 153.71 | 311.17 | 1083.42 |
| Jilin | 0.00 | 0.00 | 0.00 | 0.00 | 0.00 | 0.05 | 0.00 | 0.00 | 0.14 | 0.41 | 1.04 | 2.23 | 7.55 | 16.72 | 36.23 | 84.51 | 191.21 | 379.70 | 939.01 |
| Heilongjiang | 0.00 | 0.00 | 0.00 | 0.00 | 0.00 | 0.08 | 0.00 | 0.00 | 0.20 | 0.57 | 1.46 | 3.14 | 10.62 | 23.53 | 50.98 | 118.90 | 269.01 | 534.21 | 1321.09 |
| Shanghai | 0.00 | 0.00 | 0.00 | 0.00 | 0.00 | 0.00 | 0.00 | 0.03 | 0.13 | 0.13 | 0.69 | 1.55 | 3.57 | 12.09 | 29.15 | 73.26 | 152.75 | 309.22 | 1076.63 |
| Jiangsu | 0.00 | 0.00 | 0.00 | 0.00 | 0.00 | 0.00 | 0.00 | 0.05 | 0.19 | 0.20 | 1.06 | 2.38 | 5.49 | 18.57 | 44.78 | 112.55 | 234.66 | 475.05 | 1654.00 |
| Zhejiang | 0.00 | 0.00 | 0.00 | 0.00 | 0.00 | 0.00 | 0.00 | 0.06 | 0.22 | 0.23 | 1.21 | 2.71 | 6.25 | 21.17 | 51.03 | 128.27 | 267.43 | 541.38 | 1884.94 |
| Anhui | 0.00 | 0.00 | 0.00 | 0.00 | 0.00 | 0.11 | 0.00 | 0.00 | 0.28 | 0.81 | 2.07 | 4.46 | 15.07 | 33.39 | 72.34 | 168.73 | 381.75 | 758.10 | 1874.78 |
| Fujian | 0.00 | 0.00 | 0.00 | 0.00 | 0.00 | 0.00 | 0.00 | 0.06 | 0.23 | 0.24 | 1.27 | 2.84 | 6.54 | 22.12 | 53.33 | 134.05 | 279.47 | 565.77 | 1969.85 |
| Jiangxi | 0.00 | 0.00 | 0.00 | 0.00 | 0.00 | 0.11 | 0.00 | 0.00 | 0.30 | 0.87 | 2.22 | 4.78 | 16.16 | 35.81 | 77.59 | 180.97 | 409.45 | 813.09 | 2010.77 |
| Shandong | 0.00 | 0.00 | 0.00 | 0.00 | 0.00 | 0.00 | 0.00 | 0.05 | 0.20 | 0.21 | 1.08 | 2.43 | 5.59 | 18.92 | 45.60 | 114.63 | 239.00 | 483.83 | 1684.56 |
| Henan | 0.00 | 0.00 | 0.00 | 0.00 | 0.00 | 0.09 | 0.00 | 0.00 | 0.24 | 0.71 | 1.80 | 3.87 | 13.09 | 29.00 | 62.85 | 146.59 | 331.64 | 658.59 | 1628.69 |
| Hubei | 0.00 | 0.00 | 0.00 | 0.00 | 0.00 | 0.11 | 0.00 | 0.00 | 0.28 | 0.81 | 2.06 | 4.42 | 14.94 | 33.10 | 71.72 | 167.28 | 378.46 | 751.55 | 1858.59 |
| Hunan | 0.00 | 0.00 | 0.00 | 0.00 | 0.00 | 0.11 | 0.00 | 0.00 | 0.28 | 0.82 | 2.09 | 4.49 | 15.17 | 33.62 | 72.84 | 169.90 | 384.39 | 763.34 | 1887.73 |
| Guangdong | 0.00 | 0.00 | 0.00 | 0.00 | 0.00 | 0.00 | 0.00 | 0.05 | 0.21 | 0.22 | 1.15 | 2.57 | 5.92 | 20.02 | 48.27 | 121.34 | 252.97 | 512.12 | 1783.05 |
| Guangxi | 0.00 | 0.00 | 0.00 | 0.00 | 0.00 | 0.00 | 0.00 | 0.09 | 0.34 | 0.36 | 1.89 | 4.22 | 9.73 | 32.92 | 79.35 | 199.45 | 415.84 | 841.83 | 2931.00 |
| Hainan | 0.00 | 0.00 | 0.00 | 0.00 | 0.00 | 0.00 | 0.00 | 0.06 | 0.23 | 0.24 | 1.25 | 2.80 | 6.46 | 21.86 | 52.68 | 132.43 | 276.10 | 558.94 | 1946.08 |
| Chongqing | 0.00 | 0.00 | 0.00 | 0.00 | 0.10 | 0.00 | 0.31 | 0.35 | 0.55 | 2.90 | 5.97 | 13.13 | 27.19 | 61.94 | 142.87 | 316.92 | 745.67 | 1157.28 | 3014.09 |
| Sichuan | 0.00 | 0.00 | 0.00 | 0.00 | 0.12 | 0.00 | 0.36 | 0.41 | 0.64 | 3.37 | 6.94 | 15.25 | 31.59 | 71.98 | 166.02 | 368.27 | 866.47 | 1344.77 | 3502.39 |
| Guizhou | 0.00 | 0.00 | 0.00 | 0.00 | 0.13 | 0.00 | 0.40 | 0.45 | 0.70 | 3.68 | 7.59 | 16.68 | 34.56 | 78.73 | 181.59 | 402.80 | 947.72 | 1470.88 | 3830.83 |
| Yunnan | 0.00 | 0.00 | 0.00 | 0.00 | 0.10 | 0.00 | 0.31 | 0.36 | 0.55 | 2.91 | 6.00 | 13.19 | 27.32 | 62.24 | 143.56 | 318.45 | 749.26 | 1162.86 | 3028.62 |
| Xizang | 0.00 | 0.00 | 0.00 | 0.00 | 0.04 | 0.00 | 0.14 | 0.15 | 0.24 | 1.25 | 2.59 | 5.68 | 11.77 | 26.82 | 61.86 | 137.22 | 322.86 | 501.08 | 1305.04 |
| Shannxi | 0.00 | 0.00 | 0.00 | 0.00 | 0.04 | 0.00 | 0.13 | 0.14 | 0.22 | 1.18 | 2.43 | 5.34 | 11.06 | 25.21 | 58.14 | 128.97 | 303.44 | 470.95 | 1226.56 |
| Gansu | 0.00 | 0.00 | 0.00 | 0.00 | 0.14 | 0.00 | 0.43 | 0.48 | 0.75 | 3.94 | 8.13 | 17.86 | 36.99 | 84.28 | 194.40 | 431.23 | 1014.60 | 1574.66 | 4101.14 |
| Qinghai | 0.00 | 0.00 | 0.00 | 0.00 | 0.13 | 0.00 | 0.40 | 0.45 | 0.70 | 3.68 | 7.58 | 16.67 | 34.53 | 78.67 | 181.45 | 402.50 | 947.01 | 1469.76 | 3827.92 |
| Ningxia | 0.00 | 0.00 | 0.00 | 0.00 | 0.07 | 0.00 | 0.21 | 0.23 | 0.36 | 1.91 | 3.93 | 8.64 | 17.91 | 40.80 | 94.10 | 208.74 | 491.12 | 762.22 | 1985.17 |
| Xinjiang | 0.00 | 0.00 | 0.00 | 0.00 | 0.10 | 0.00 | 0.30 | 0.34 | 0.52 | 2.77 | 5.71 | 12.54 | 25.98 | 59.20 | 136.54 | 302.87 | 712.59 | 1105.95 | 2880.39 |

# Table S7 Baseline death rates of females for different age groups in different provinces.

|  | **0-1** | **1-5** | **5-10** | **10-15** | **15-20** | **20-25** | **25-30** | **30-35** | **35-40** | **40-45** | **45-50** | **50-55** | **55-60** | **60-65** | **65-70** | **70-75** | **75-80** | **80-85** | **>85** |
| --- | --- | --- | --- | --- | --- | --- | --- | --- | --- | --- | --- | --- | --- | --- | --- | --- | --- | --- | --- |
| Beijing | 0.00 | 0.00 | 0.00 | 0.00 | 0.00 | 0.00 | 0.00 | 0.00 | 0.09 | 0.09 | 0.63 | 1.41 | 2.42 | 7.77 | 21.42 | 55.91 | 124.08 | 331.46 | 1370.09 |
| Tianjin | 0.00 | 0.00 | 0.00 | 0.00 | 0.00 | 0.00 | 0.00 | 0.00 | 0.08 | 0.08 | 0.57 | 1.27 | 2.18 | 7.01 | 19.31 | 50.43 | 111.90 | 298.93 | 1235.63 |
| Hebei | 0.00 | 0.00 | 0.00 | 0.00 | 0.00 | 0.00 | 0.00 | 0.00 | 0.12 | 0.12 | 0.82 | 1.83 | 3.15 | 10.12 | 27.89 | 72.82 | 161.60 | 431.69 | 1784.39 |
| Shanxi | 0.00 | 0.00 | 0.00 | 0.00 | 0.00 | 0.00 | 0.00 | 0.36 | 0.61 | 0.59 | 1.15 | 1.97 | 10.04 | 20.77 | 55.73 | 125.01 | 320.71 | 815.47 | 2547.93 |
| Inner Mongolia | 0.00 | 0.00 | 0.00 | 0.00 | 0.00 | 0.00 | 0.00 | 0.40 | 0.68 | 0.65 | 1.28 | 2.19 | 11.15 | 23.07 | 61.92 | 138.88 | 356.29 | 905.95 | 2830.65 |
| Liaoning | 0.00 | 0.00 | 0.00 | 0.00 | 0.00 | 0.00 | 0.00 | 0.00 | 0.11 | 0.11 | 0.73 | 1.62 | 2.78 | 8.94 | 24.65 | 64.37 | 142.84 | 381.58 | 1577.24 |
| Jilin | 0.00 | 0.00 | 0.00 | 0.00 | 0.00 | 0.00 | 0.00 | 0.19 | 0.33 | 0.32 | 0.63 | 1.07 | 5.46 | 11.29 | 30.30 | 67.97 | 174.37 | 443.36 | 1385.28 |
| Heilongjiang | 0.00 | 0.00 | 0.00 | 0.00 | 0.00 | 0.00 | 0.00 | 0.25 | 0.42 | 0.41 | 0.80 | 1.37 | 6.96 | 14.40 | 38.65 | 86.69 | 222.41 | 565.51 | 1766.94 |
| Shanghai | 0.00 | 0.00 | 0.00 | 0.00 | 0.00 | 0.00 | 0.00 | 0.00 | 0.17 | 0.17 | 1.14 | 2.54 | 4.38 | 14.06 | 38.74 | 101.15 | 224.47 | 599.62 | 2478.52 |
| Jiangsu | 0.00 | 0.00 | 0.00 | 0.00 | 0.00 | 0.00 | 0.00 | 0.00 | 0.20 | 0.20 | 1.35 | 3.02 | 5.19 | 16.67 | 45.96 | 119.99 | 266.27 | 711.28 | 2940.06 |
| Zhejiang | 0.00 | 0.00 | 0.00 | 0.00 | 0.00 | 0.00 | 0.00 | 0.00 | 0.21 | 0.21 | 1.45 | 3.24 | 5.58 | 17.91 | 49.37 | 128.88 | 286.01 | 764.04 | 3158.12 |
| Anhui | 0.00 | 0.00 | 0.00 | 0.00 | 0.00 | 0.00 | 0.00 | 0.45 | 0.77 | 0.74 | 1.46 | 2.50 | 12.70 | 26.27 | 70.50 | 158.13 | 405.67 | 1031.49 | 3222.91 |
| Fujian | 0.00 | 0.00 | 0.00 | 0.00 | 0.00 | 0.00 | 0.00 | 0.00 | 0.29 | 0.29 | 2.01 | 4.47 | 7.69 | 24.71 | 68.11 | 177.83 | 394.63 | 1054.18 | 4357.40 |
| Jiangxi | 0.00 | 0.00 | 0.00 | 0.00 | 0.00 | 0.00 | 0.00 | 0.52 | 0.88 | 0.85 | 1.67 | 2.85 | 14.49 | 29.99 | 80.47 | 180.50 | 463.05 | 1177.39 | 3678.78 |
| Shandong | 0.00 | 0.00 | 0.00 | 0.00 | 0.00 | 0.00 | 0.00 | 0.00 | 0.19 | 0.19 | 1.34 | 2.98 | 5.12 | 16.45 | 45.33 | 118.35 | 262.65 | 701.61 | 2900.09 |
| Henan | 0.00 | 0.00 | 0.00 | 0.00 | 0.00 | 0.00 | 0.00 | 0.44 | 0.76 | 0.73 | 1.43 | 2.45 | 12.46 | 25.78 | 69.18 | 155.18 | 398.11 | 1012.26 | 3162.83 |
| Hubei | 0.00 | 0.00 | 0.00 | 0.00 | 0.00 | 0.00 | 0.00 | 0.50 | 0.86 | 0.83 | 1.63 | 2.78 | 14.14 | 29.27 | 78.54 | 176.16 | 451.93 | 1149.12 | 3590.43 |
| Hunan | 0.00 | 0.00 | 0.00 | 0.00 | 0.00 | 0.00 | 0.00 | 0.48 | 0.83 | 0.80 | 1.57 | 2.68 | 13.63 | 28.20 | 75.68 | 169.75 | 435.47 | 1107.27 | 3459.68 |
| Guangdong | 0.00 | 0.00 | 0.00 | 0.00 | 0.00 | 0.00 | 0.00 | 0.00 | 0.23 | 0.23 | 1.60 | 3.57 | 6.14 | 19.72 | 54.36 | 141.94 | 314.98 | 841.41 | 3477.92 |
| Guangxi | 0.00 | 0.00 | 0.00 | 0.00 | 0.00 | 0.00 | 0.00 | 0.00 | 0.33 | 0.33 | 2.24 | 5.00 | 8.60 | 27.62 | 76.12 | 198.74 | 441.03 | 1178.14 | 4869.82 |
| Hainan | 0.00 | 0.00 | 0.00 | 0.00 | 0.00 | 0.00 | 0.00 | 0.00 | 0.25 | 0.25 | 1.70 | 3.79 | 6.52 | 20.94 | 57.72 | 150.69 | 334.40 | 893.28 | 3692.34 |
| Chongqing | 0.00 | 0.00 | 0.00 | 0.00 | 0.00 | 0.29 | 0.44 | 1.12 | 0.70 | 2.50 | 5.27 | 13.62 | 19.75 | 59.35 | 150.07 | 404.59 | 961.61 | 1871.78 | 5317.92 |
| Sichuan | 0.00 | 0.00 | 0.00 | 0.00 | 0.00 | 0.29 | 0.44 | 1.13 | 0.71 | 2.53 | 5.33 | 13.76 | 19.95 | 59.93 | 151.55 | 408.59 | 971.13 | 1890.32 | 5370.57 |
| Guizhou | 0.00 | 0.00 | 0.00 | 0.00 | 0.00 | 0.33 | 0.50 | 1.28 | 0.80 | 2.86 | 6.02 | 15.55 | 22.55 | 67.74 | 171.30 | 461.84 | 1097.70 | 2136.69 | 6070.55 |
| Yunnan | 0.00 | 0.00 | 0.00 | 0.00 | 0.00 | 0.29 | 0.45 | 1.15 | 0.72 | 2.56 | 5.39 | 13.93 | 20.20 | 60.69 | 153.47 | 413.78 | 983.45 | 1914.30 | 5438.71 |
| Xizang | 0.00 | 0.00 | 0.00 | 0.00 | 0.00 | 0.11 | 0.16 | 0.42 | 0.26 | 0.93 | 1.95 | 5.05 | 7.32 | 21.98 | 55.59 | 149.86 | 356.19 | 693.33 | 1969.83 |
| Shannxi | 0.00 | 0.00 | 0.00 | 0.00 | 0.00 | 0.11 | 0.17 | 0.45 | 0.28 | 0.99 | 2.09 | 5.41 | 7.85 | 23.57 | 59.61 | 160.70 | 381.96 | 743.48 | 2112.30 |
| Gansu | 0.00 | 0.00 | 0.00 | 0.00 | 0.00 | 0.29 | 0.45 | 1.14 | 0.72 | 2.56 | 5.38 | 13.91 | 20.17 | 60.59 | 153.21 | 413.07 | 981.77 | 1911.03 | 5429.42 |
| Qinghai | 0.00 | 0.00 | 0.00 | 0.00 | 0.00 | 0.27 | 0.42 | 1.06 | 0.67 | 2.37 | 5.00 | 12.92 | 18.73 | 56.27 | 142.29 | 383.61 | 911.77 | 1774.76 | 5042.27 |
| Ningxia | 0.00 | 0.00 | 0.00 | 0.00 | 0.00 | 0.13 | 0.21 | 0.53 | 0.33 | 1.18 | 2.48 | 6.39 | 9.27 | 27.86 | 70.44 | 189.92 | 451.40 | 878.66 | 2496.36 |
| Xinjiang | 0.00 | 0.00 | 0.00 | 0.00 | 0.00 | 0.20 | 0.31 | 0.78 | 0.49 | 1.75 | 3.68 | 9.50 | 13.77 | 41.37 | 104.62 | 282.06 | 670.38 | 1304.91 | 3707.37 |

# Table S8 Proportion for populations with different ages for male in different provinces and municipalities. (Values for a province are used for all the cities in that province.)

| **Province** | **0-1** | **1-5** | **5-10** | **10-15** | **15-20** | **20-25** | **25-30** | **30-35** | **35-40** | **40-45** | **45-50** | **50-55** | **55-60** | **60-65** | **65-70** | **70-75** | **75-80** | **80-85** | **>85** |
| --- | --- | --- | --- | --- | --- | --- | --- | --- | --- | --- | --- | --- | --- | --- | --- | --- | --- | --- | --- |
| Beijing | 0.004 | 0.020 | 0.018 | 0.018 | 0.025 | 0.061 | 0.054 | 0.042 | 0.041 | 0.039 | 0.038 | 0.031 | 0.027 | 0.016 | 0.020 | 0.019 | 0.015 | 0.008 | 0.004 |
| Tianjin | 0.004 | 0.020 | 0.021 | 0.022 | 0.027 | 0.058 | 0.047 | 0.037 | 0.037 | 0.037 | 0.037 | 0.033 | 0.029 | 0.018 | 0.025 | 0.020 | 0.016 | 0.009 | 0.004 |
| Hebei | 0.007 | 0.033 | 0.034 | 0.027 | 0.033 | 0.046 | 0.036 | 0.030 | 0.032 | 0.039 | 0.034 | 0.030 | 0.029 | 0.021 | 0.026 | 0.020 | 0.014 | 0.007 | 0.003 |
| Shanxi | 0.005 | 0.020 | 0.025 | 0.032 | 0.044 | 0.042 | 0.036 | 0.033 | 0.043 | 0.042 | 0.038 | 0.031 | 0.027 | 0.019 | 0.023 | 0.019 | 0.013 | 0.006 | 0.003 |
| Inner Mongolia | 0.004 | 0.019 | 0.022 | 0.025 | 0.031 | 0.039 | 0.039 | 0.038 | 0.048 | 0.048 | 0.042 | 0.034 | 0.028 | 0.018 | 0.023 | 0.021 | 0.013 | 0.006 | 0.002 |
| Liaoning | 0.003 | 0.014 | 0.018 | 0.020 | 0.027 | 0.038 | 0.031 | 0.034 | 0.039 | 0.044 | 0.045 | 0.040 | 0.036 | 0.023 | 0.030 | 0.024 | 0.017 | 0.010 | 0.005 |
| Jilin | 0.003 | 0.016 | 0.019 | 0.020 | 0.028 | 0.041 | 0.032 | 0.036 | 0.043 | 0.049 | 0.044 | 0.036 | 0.033 | 0.021 | 0.028 | 0.023 | 0.015 | 0.008 | 0.004 |
| Heilongjiang | 0.003 | 0.013 | 0.017 | 0.019 | 0.028 | 0.040 | 0.034 | 0.037 | 0.048 | 0.049 | 0.045 | 0.037 | 0.032 | 0.021 | 0.028 | 0.024 | 0.015 | 0.008 | 0.004 |
| Shanghai | 0.003 | 0.016 | 0.016 | 0.014 | 0.022 | 0.051 | 0.050 | 0.042 | 0.039 | 0.038 | 0.036 | 0.035 | 0.033 | 0.022 | 0.026 | 0.019 | 0.019 | 0.011 | 0.007 |
| Jiangsu | 0.005 | 0.023 | 0.024 | 0.024 | 0.034 | 0.044 | 0.032 | 0.031 | 0.036 | 0.045 | 0.037 | 0.029 | 0.031 | 0.023 | 0.029 | 0.023 | 0.016 | 0.009 | 0.004 |
| Zhejiang | 0.004 | 0.019 | 0.022 | 0.022 | 0.032 | 0.043 | 0.039 | 0.039 | 0.045 | 0.047 | 0.041 | 0.031 | 0.029 | 0.021 | 0.021 | 0.018 | 0.015 | 0.008 | 0.004 |
| Anhui | 0.007 | 0.027 | 0.030 | 0.032 | 0.038 | 0.036 | 0.027 | 0.031 | 0.041 | 0.047 | 0.038 | 0.020 | 0.028 | 0.023 | 0.029 | 0.021 | 0.014 | 0.007 | 0.003 |
| Fujian | 0.007 | 0.030 | 0.031 | 0.029 | 0.036 | 0.047 | 0.040 | 0.038 | 0.044 | 0.042 | 0.034 | 0.026 | 0.024 | 0.016 | 0.019 | 0.016 | 0.011 | 0.006 | 0.003 |
| Jiangxi | 0.006 | 0.033 | 0.037 | 0.034 | 0.038 | 0.042 | 0.031 | 0.037 | 0.042 | 0.040 | 0.032 | 0.026 | 0.024 | 0.018 | 0.021 | 0.018 | 0.011 | 0.006 | 0.003 |
| Shandong | 0.006 | 0.028 | 0.031 | 0.029 | 0.026 | 0.044 | 0.032 | 0.030 | 0.037 | 0.043 | 0.037 | 0.029 | 0.031 | 0.022 | 0.027 | 0.021 | 0.015 | 0.008 | 0.004 |
| Henan | 0.006 | 0.036 | 0.038 | 0.036 | 0.037 | 0.045 | 0.028 | 0.028 | 0.036 | 0.039 | 0.032 | 0.023 | 0.027 | 0.020 | 0.026 | 0.019 | 0.013 | 0.007 | 0.003 |
| Hubei | 0.006 | 0.024 | 0.025 | 0.026 | 0.039 | 0.043 | 0.031 | 0.030 | 0.038 | 0.044 | 0.040 | 0.029 | 0.029 | 0.022 | 0.028 | 0.021 | 0.014 | 0.007 | 0.003 |
| Hunan | 0.007 | 0.029 | 0.033 | 0.029 | 0.029 | 0.041 | 0.031 | 0.029 | 0.040 | 0.044 | 0.037 | 0.026 | 0.028 | 0.022 | 0.026 | 0.022 | 0.015 | 0.008 | 0.004 |
| Guangdong | 0.006 | 0.024 | 0.028 | 0.036 | 0.046 | 0.055 | 0.049 | 0.041 | 0.044 | 0.040 | 0.032 | 0.022 | 0.020 | 0.014 | 0.014 | 0.012 | 0.009 | 0.005 | 0.003 |
| Guangxi | 0.009 | 0.033 | 0.039 | 0.037 | 0.035 | 0.039 | 0.036 | 0.035 | 0.038 | 0.039 | 0.033 | 0.024 | 0.024 | 0.018 | 0.021 | 0.017 | 0.012 | 0.006 | 0.004 |
| Hainan | 0.007 | 0.028 | 0.031 | 0.034 | 0.044 | 0.044 | 0.042 | 0.038 | 0.040 | 0.041 | 0.038 | 0.025 | 0.022 | 0.016 | 0.016 | 0.016 | 0.011 | 0.006 | 0.003 |
| Chongqing | 0.004 | 0.021 | 0.025 | 0.029 | 0.037 | 0.034 | 0.025 | 0.023 | 0.046 | 0.045 | 0.036 | 0.026 | 0.036 | 0.028 | 0.034 | 0.024 | 0.015 | 0.009 | 0.004 |
| Sichuan | 0.004 | 0.021 | 0.025 | 0.030 | 0.036 | 0.036 | 0.026 | 0.028 | 0.047 | 0.045 | 0.034 | 0.024 | 0.033 | 0.025 | 0.033 | 0.024 | 0.015 | 0.009 | 0.004 |
| Guizhou | 0.007 | 0.028 | 0.038 | 0.048 | 0.041 | 0.033 | 0.028 | 0.034 | 0.044 | 0.042 | 0.033 | 0.022 | 0.025 | 0.020 | 0.022 | 0.018 | 0.011 | 0.005 | 0.002 |
| Yunnan | 0.006 | 0.024 | 0.032 | 0.036 | 0.039 | 0.043 | 0.038 | 0.042 | 0.047 | 0.044 | 0.035 | 0.022 | 0.022 | 0.016 | 0.020 | 0.016 | 0.010 | 0.005 | 0.002 |
| Xizang | 0.008 | 0.034 | 0.040 | 0.041 | 0.045 | 0.055 | 0.051 | 0.042 | 0.041 | 0.038 | 0.031 | 0.020 | 0.015 | 0.012 | 0.012 | 0.008 | 0.005 | 0.002 | 0.001 |
| Shannxi | 0.005 | 0.023 | 0.026 | 0.032 | 0.041 | 0.047 | 0.034 | 0.031 | 0.040 | 0.041 | 0.036 | 0.030 | 0.027 | 0.019 | 0.026 | 0.020 | 0.012 | 0.006 | 0.003 |
| Gansu | 0.006 | 0.024 | 0.030 | 0.037 | 0.045 | 0.041 | 0.030 | 0.029 | 0.043 | 0.048 | 0.037 | 0.024 | 0.025 | 0.019 | 0.026 | 0.020 | 0.011 | 0.004 | 0.002 |
| Qinghai | 0.006 | 0.026 | 0.034 | 0.037 | 0.042 | 0.042 | 0.036 | 0.042 | 0.050 | 0.049 | 0.037 | 0.021 | 0.020 | 0.014 | 0.018 | 0.014 | 0.008 | 0.003 | 0.001 |
| Ningxia | 0.006 | 0.026 | 0.033 | 0.037 | 0.042 | 0.042 | 0.038 | 0.042 | 0.046 | 0.045 | 0.035 | 0.023 | 0.022 | 0.016 | 0.019 | 0.015 | 0.009 | 0.004 | 0.002 |
| Xinjiang | 0.008 | 0.031 | 0.035 | 0.038 | 0.038 | 0.046 | 0.039 | 0.039 | 0.049 | 0.048 | 0.035 | 0.023 | 0.018 | 0.015 | 0.014 | 0.013 | 0.007 | 0.003 | 0.002 |

# Table S9 Proportion for populations with different ages for females in different provinces and municipalities. (Values for a province are used for all the cities in that province.)

| **Province** | **0-1** | **1-5** | **5-10** | **10-15** | **15-20** | **20-25** | **25-30** | **30-35** | **35-40** | **40-45** | **45-50** | **50-55** | **55-60** | **60-65** | **65-70** | **70-75** | **75-80** | **80-85** | **>85** |
| --- | --- | --- | --- | --- | --- | --- | --- | --- | --- | --- | --- | --- | --- | --- | --- | --- | --- | --- | --- |
| Beijing | 0.004 | 0.020 | 0.017 | 0.018 | 0.024 | 0.061 | 0.056 | 0.042 | 0.039 | 0.037 | 0.037 | 0.032 | 0.029 | 0.018 | 0.020 | 0.020 | 0.015 | 0.008 | 0.004 |
| Tianjin | 0.004 | 0.020 | 0.021 | 0.022 | 0.028 | 0.056 | 0.044 | 0.034 | 0.034 | 0.036 | 0.038 | 0.035 | 0.033 | 0.021 | 0.024 | 0.020 | 0.015 | 0.009 | 0.005 |
| Hebei | 0.007 | 0.032 | 0.034 | 0.028 | 0.032 | 0.047 | 0.037 | 0.029 | 0.032 | 0.038 | 0.035 | 0.029 | 0.029 | 0.021 | 0.024 | 0.019 | 0.014 | 0.008 | 0.005 |
| Shanxi | 0.005 | 0.020 | 0.025 | 0.033 | 0.045 | 0.042 | 0.036 | 0.033 | 0.043 | 0.042 | 0.037 | 0.030 | 0.027 | 0.019 | 0.022 | 0.018 | 0.014 | 0.007 | 0.004 |
| Inner Mongolia | 0.004 | 0.018 | 0.022 | 0.026 | 0.032 | 0.040 | 0.039 | 0.036 | 0.047 | 0.047 | 0.042 | 0.034 | 0.029 | 0.019 | 0.024 | 0.020 | 0.013 | 0.006 | 0.003 |
| Liaoning | 0.003 | 0.014 | 0.018 | 0.020 | 0.026 | 0.038 | 0.032 | 0.034 | 0.039 | 0.043 | 0.044 | 0.040 | 0.037 | 0.024 | 0.029 | 0.024 | 0.018 | 0.010 | 0.006 |
| Jilin | 0.003 | 0.016 | 0.019 | 0.020 | 0.028 | 0.042 | 0.033 | 0.036 | 0.042 | 0.047 | 0.043 | 0.036 | 0.034 | 0.023 | 0.029 | 0.023 | 0.015 | 0.008 | 0.004 |
| Heilongjiang | 0.003 | 0.013 | 0.017 | 0.019 | 0.027 | 0.041 | 0.034 | 0.037 | 0.047 | 0.048 | 0.044 | 0.037 | 0.033 | 0.023 | 0.029 | 0.024 | 0.014 | 0.007 | 0.004 |
| Shanghai | 0.003 | 0.016 | 0.015 | 0.014 | 0.022 | 0.053 | 0.052 | 0.042 | 0.037 | 0.036 | 0.035 | 0.036 | 0.035 | 0.023 | 0.021 | 0.018 | 0.020 | 0.013 | 0.010 |
| Jiangsu | 0.005 | 0.023 | 0.024 | 0.024 | 0.031 | 0.045 | 0.033 | 0.032 | 0.037 | 0.046 | 0.038 | 0.028 | 0.031 | 0.023 | 0.025 | 0.021 | 0.017 | 0.011 | 0.008 |
| Zhejiang | 0.004 | 0.019 | 0.022 | 0.022 | 0.031 | 0.043 | 0.039 | 0.039 | 0.045 | 0.048 | 0.041 | 0.030 | 0.029 | 0.022 | 0.019 | 0.016 | 0.015 | 0.009 | 0.006 |
| Anhui | 0.007 | 0.027 | 0.030 | 0.033 | 0.035 | 0.039 | 0.029 | 0.031 | 0.041 | 0.048 | 0.038 | 0.019 | 0.028 | 0.021 | 0.026 | 0.019 | 0.015 | 0.009 | 0.006 |
| Fujian | 0.007 | 0.029 | 0.032 | 0.029 | 0.034 | 0.049 | 0.041 | 0.037 | 0.044 | 0.042 | 0.034 | 0.026 | 0.024 | 0.016 | 0.016 | 0.014 | 0.012 | 0.008 | 0.005 |
| Jiangxi | 0.007 | 0.032 | 0.036 | 0.035 | 0.034 | 0.043 | 0.033 | 0.039 | 0.042 | 0.040 | 0.032 | 0.026 | 0.024 | 0.018 | 0.019 | 0.017 | 0.012 | 0.007 | 0.005 |
| Shandong | 0.006 | 0.026 | 0.031 | 0.030 | 0.024 | 0.044 | 0.032 | 0.030 | 0.037 | 0.043 | 0.038 | 0.029 | 0.031 | 0.022 | 0.024 | 0.019 | 0.016 | 0.010 | 0.007 |
| Henan | 0.006 | 0.035 | 0.037 | 0.036 | 0.034 | 0.047 | 0.030 | 0.029 | 0.035 | 0.040 | 0.033 | 0.023 | 0.027 | 0.019 | 0.023 | 0.017 | 0.014 | 0.008 | 0.006 |
| Hubei | 0.006 | 0.025 | 0.025 | 0.026 | 0.036 | 0.045 | 0.033 | 0.031 | 0.038 | 0.044 | 0.040 | 0.029 | 0.029 | 0.021 | 0.026 | 0.020 | 0.015 | 0.008 | 0.005 |
| Hunan | 0.007 | 0.029 | 0.033 | 0.030 | 0.028 | 0.044 | 0.034 | 0.030 | 0.039 | 0.044 | 0.037 | 0.025 | 0.028 | 0.021 | 0.023 | 0.021 | 0.015 | 0.009 | 0.006 |
| Guangdong | 0.006 | 0.024 | 0.027 | 0.037 | 0.045 | 0.057 | 0.048 | 0.040 | 0.043 | 0.041 | 0.032 | 0.022 | 0.020 | 0.014 | 0.012 | 0.011 | 0.009 | 0.006 | 0.005 |
| Guangxi | 0.008 | 0.033 | 0.039 | 0.037 | 0.034 | 0.039 | 0.037 | 0.035 | 0.037 | 0.038 | 0.033 | 0.024 | 0.025 | 0.018 | 0.019 | 0.016 | 0.013 | 0.008 | 0.006 |
| Hainan | 0.007 | 0.028 | 0.031 | 0.033 | 0.043 | 0.046 | 0.044 | 0.038 | 0.039 | 0.040 | 0.036 | 0.024 | 0.022 | 0.016 | 0.013 | 0.014 | 0.011 | 0.007 | 0.006 |
| Chongqing | 0.004 | 0.021 | 0.025 | 0.029 | 0.036 | 0.036 | 0.026 | 0.024 | 0.047 | 0.044 | 0.036 | 0.024 | 0.036 | 0.026 | 0.030 | 0.022 | 0.016 | 0.010 | 0.006 |
| Sichuan | 0.004 | 0.021 | 0.025 | 0.030 | 0.035 | 0.037 | 0.027 | 0.029 | 0.047 | 0.045 | 0.034 | 0.023 | 0.033 | 0.024 | 0.030 | 0.023 | 0.016 | 0.010 | 0.006 |
| Guizhou | 0.006 | 0.026 | 0.037 | 0.050 | 0.039 | 0.035 | 0.030 | 0.034 | 0.043 | 0.041 | 0.032 | 0.022 | 0.026 | 0.020 | 0.021 | 0.017 | 0.011 | 0.006 | 0.003 |
| Yunnan | 0.006 | 0.024 | 0.032 | 0.036 | 0.039 | 0.044 | 0.038 | 0.041 | 0.045 | 0.043 | 0.035 | 0.023 | 0.023 | 0.017 | 0.018 | 0.015 | 0.011 | 0.006 | 0.003 |
| Xizang | 0.008 | 0.034 | 0.040 | 0.042 | 0.045 | 0.053 | 0.051 | 0.041 | 0.040 | 0.037 | 0.031 | 0.020 | 0.016 | 0.014 | 0.011 | 0.008 | 0.005 | 0.003 | 0.002 |
| Shannxi | 0.005 | 0.023 | 0.026 | 0.032 | 0.039 | 0.048 | 0.034 | 0.031 | 0.040 | 0.041 | 0.036 | 0.030 | 0.028 | 0.020 | 0.025 | 0.020 | 0.013 | 0.006 | 0.003 |
| Gansu | 0.006 | 0.023 | 0.030 | 0.038 | 0.043 | 0.043 | 0.031 | 0.029 | 0.043 | 0.048 | 0.037 | 0.022 | 0.024 | 0.020 | 0.025 | 0.019 | 0.012 | 0.004 | 0.002 |
| Qinghai | 0.006 | 0.026 | 0.034 | 0.038 | 0.043 | 0.042 | 0.037 | 0.042 | 0.048 | 0.047 | 0.036 | 0.021 | 0.020 | 0.016 | 0.018 | 0.013 | 0.008 | 0.003 | 0.002 |
| Ningxia | 0.006 | 0.026 | 0.033 | 0.038 | 0.043 | 0.043 | 0.039 | 0.041 | 0.045 | 0.044 | 0.034 | 0.022 | 0.023 | 0.016 | 0.019 | 0.015 | 0.009 | 0.004 | 0.002 |
| Xinjiang | 0.008 | 0.031 | 0.035 | 0.038 | 0.039 | 0.047 | 0.041 | 0.040 | 0.048 | 0.046 | 0.032 | 0.022 | 0.019 | 0.016 | 0.016 | 0.012 | 0.006 | 0.003 | 0.002 |

# Table S10 Proportion of the populations with different ages in different provinces and municipalities under different scenarios. (Values for a province are used for all the cities in that province.)

|  | **2019** | | | **2030** | | | **2050** | | |
| --- | --- | --- | --- | --- | --- | --- | --- | --- | --- |
| **Age** | **0-15** | **15-65** | **>65** | **0-15** | **15-65** | **>65** | **0-15** | **15-65** | **>65** |
| Beijing | 0.12 | 0.75 | 0.13 | 0.08 | 0.71 | 0.21 | 0.07 | 0.54 | 0.38 |
| Tianjin | 0.13 | 0.72 | 0.15 | 0.09 | 0.69 | 0.21 | 0.08 | 0.56 | 0.36 |
| Hebei | 0.20 | 0.66 | 0.14 | 0.14 | 0.67 | 0.19 | 0.13 | 0.61 | 0.26 |
| Shanxi | 0.16 | 0.71 | 0.13 | 0.12 | 0.70 | 0.18 | 0.10 | 0.61 | 0.28 |
| InnerMongolia | 0.14 | 0.73 | 0.13 | 0.09 | 0.69 | 0.22 | 0.09 | 0.55 | 0.37 |
| Liaoning | 0.11 | 0.71 | 0.17 | 0.07 | 0.64 | 0.29 | 0.06 | 0.49 | 0.45 |
| Jilin | 0.12 | 0.73 | 0.16 | 0.08 | 0.66 | 0.26 | 0.07 | 0.49 | 0.44 |
| Heilongjiang | 0.10 | 0.74 | 0.16 | 0.07 | 0.66 | 0.27 | 0.06 | 0.47 | 0.47 |
| Shanghai | 0.10 | 0.74 | 0.16 | 0.07 | 0.69 | 0.24 | 0.06 | 0.53 | 0.41 |
| Jiangsu | 0.15 | 0.69 | 0.16 | 0.11 | 0.68 | 0.22 | 0.10 | 0.58 | 0.32 |
| Zhejiang | 0.13 | 0.73 | 0.13 | 0.09 | 0.69 | 0.21 | 0.08 | 0.56 | 0.36 |
| Anhui | 0.19 | 0.66 | 0.15 | 0.14 | 0.68 | 0.18 | 0.13 | 0.60 | 0.28 |
| Fujian | 0.19 | 0.70 | 0.11 | 0.14 | 0.70 | 0.16 | 0.12 | 0.61 | 0.27 |
| Jiangxi | 0.22 | 0.66 | 0.12 | 0.16 | 0.68 | 0.16 | 0.14 | 0.61 | 0.25 |
| Shandong | 0.19 | 0.66 | 0.15 | 0.13 | 0.65 | 0.22 | 0.12 | 0.58 | 0.30 |
| Henan | 0.23 | 0.63 | 0.13 | 0.17 | 0.66 | 0.17 | 0.15 | 0.62 | 0.23 |
| Hubei | 0.16 | 0.69 | 0.15 | 0.11 | 0.68 | 0.21 | 0.10 | 0.62 | 0.28 |
| Hunan | 0.20 | 0.66 | 0.15 | 0.14 | 0.66 | 0.20 | 0.12 | 0.57 | 0.30 |
| Guangdong | 0.19 | 0.73 | 0.09 | 0.13 | 0.73 | 0.14 | 0.12 | 0.60 | 0.28 |
| Guangxi | 0.24 | 0.64 | 0.12 | 0.17 | 0.68 | 0.15 | 0.15 | 0.62 | 0.23 |
| Hainan | 0.20 | 0.70 | 0.10 | 0.14 | 0.72 | 0.14 | 0.13 | 0.63 | 0.24 |
| Chongqing | 0.16 | 0.67 | 0.17 | 0.11 | 0.66 | 0.23 | 0.10 | 0.58 | 0.32 |
| Sichuan | 0.16 | 0.67 | 0.17 | 0.12 | 0.68 | 0.21 | 0.10 | 0.60 | 0.29 |
| Guizhou | 0.24 | 0.64 | 0.12 | 0.17 | 0.68 | 0.14 | 0.16 | 0.63 | 0.22 |
| Yunnan | 0.20 | 0.70 | 0.11 | 0.14 | 0.73 | 0.13 | 0.13 | 0.65 | 0.22 |
| Tibet | 0.25 | 0.70 | 0.06 | 0.18 | 0.73 | 0.10 | 0.16 | 0.65 | 0.20 |
| Shaanxi | 0.17 | 0.69 | 0.13 | 0.12 | 0.69 | 0.19 | 0.11 | 0.64 | 0.25 |
| Gansu | 0.19 | 0.68 | 0.13 | 0.14 | 0.70 | 0.16 | 0.13 | 0.62 | 0.25 |
| Qinghai | 0.21 | 0.71 | 0.09 | 0.15 | 0.73 | 0.12 | 0.14 | 0.62 | 0.24 |
| Ningxia | 0.20 | 0.70 | 0.10 | 0.15 | 0.72 | 0.13 | 0.13 | 0.62 | 0.24 |
| Xinjiang | 0.22 | 0.70 | 0.08 | 0.16 | 0.71 | 0.13 | 0.14 | 0.62 | 0.24 |

# Table S11: Information regarding the model evaluation cases and the parameter settings for the model.

| Case | Case | Period | City | Population |
| --- | --- | --- | --- | --- |
| Case 1 | Field study 1 | 5/29/2016–12/12/2016 | Shanghai | College students |
|  | Model 1 | 5/29/2017–12/12/2017 | Shanghai | 18 to 44 yrs old |
| Case 2 | Field study 2 | 10/31/2017–11/3/2017 | Nanjing | Over 50 yrs old |
|  | Model 2 | 10/31/2017–11/3/2017 | Nanjing | 45 to 79 yrs old |
| Case 3 | Field study 3 | 12/2/2014–1/30/2015 | Changsha | 22 to 52 yrs old |
|  | Model 3 | 12/2/2014–1/30/2015 | Changsha | 18 to 59 yrs old |
| Case 4 | Field study 4 | 2/14/2017-4/27/2017 | Shanghai | 5 to 14 yrs old |
|  | Model 4 | 2/14/2017-4/27/2017 | Shanghai | 5 to 17 yrs old |

Field study 1: Niu et al. (2018); Field study 2: Zhang et al. (2019)

# References

[1] M. Yao, C.J. Weschler, B. Zhao, L. Zhang, R. Ma, Breathing-rate adjusted population exposure to ozone and its oxidation products in 333 cities in China, Environ Int 138 (2020) 105617.

[2] M. Yao, L. Ke, Y. Liu, Z. Luo, B. Zhao, Measurement of ozone deposition velocity onto human surfaces of Chinese residents and estimation of corresponding production of oxidation products, Environ Pollut 266(Pt 3) (2020) 115215.

[3] M.Y. Yao, B. Zhao, Surface removal rate of ozone in residences in China, Building and Environment 142 (2018) 101-106.

[4] Y. Hu, B. Zhao, Indoor sources strongly contribute to exposure of Chinese urban residents to PM2.5 and NO2, J Hazard Mater 426 (2022) 127829.

[5] M. Yao, B. Zhao, Window opening behavior of occupants in residential buildings in Beijing, Building and Environment 124 (2017) 441-449.

[6] C.O.o.t.S. Council, N.B.o. Statistics, P.a.E.S. Division, Tabulation on the 2010 population census of the People's Republic of China, China Statistics Press, Beijing, 2012.

[7] L.Y. Duan, Z.Y. Liu, W. Yu, W. Chen, D.Y. Jin, Y. Feng, Y. Wang, J.J. Liu, H. Zhou, S.H. Sun, et al., The provincial trend of population aging in China - based on population expansion forecast formula, J Comput Methods Sci 22(1) (2022) 349-359.

[8] Q. Li, M. Reuser, C. Kraus, J. Alho, Ageing of a giant: a stochastic population forecast for China, 2006–2060, Journal of Population Research 26(1) (2009) 21-50.

[9] Y. Niu, J. Cai, Y. Xia, H. Yu, R. Chen, Z. Lin, C. Liu, C. Chen, W. Wang, L. Peng, et al., Estimation of personal ozone exposure using ambient concentrations and influencing factors, Environment International 117 (2018) 237-242.

[10] J. Zhang, H. Sun, Q. Chen, J. Gu, Z. Ding, Y. Xu, Effects of individual ozone exposure on lung function in the elderly: a cross-sectional study in China, Environmental science and pollution research international 26(12) (2019) 11690-11695.

[11] D.B. Day, J. Xiang, J. Mo, F. Li, M. Chung, J. Gong, C.J. Weschler, P.A. Ohman-Strickland, J. Sundell, W. Weng, et al., Association of Ozone Exposure With Cardiorespiratory Pathophysiologic Mechanisms in Healthy Adults, JAMA Intern Med 177(9) (2017) 1344-1353.

[12] K.K. Barkjohn, C. Norris, X. Cui, L. Fang, L. He, J.J. Schauer, Y. Zhang, M. Black, J. Zhang, M.H. Bergin, Children’s microenvironmental exposure to PM2.5 and ozone and the impact of indoor air filtration, Journal of Exposure Science & Environmental Epidemiology 30(6) (2020) 971-980.

[13] J. Shen, Research on source,removal and dispersion of indoor ozone, Nanjing University, 2018, p. 127.
